# Putin, der Cliotherapeut. Überdosis an Geschichte und politisierte Erinnerungskonflikte in Osteuropa

**DOI:** 10.1007/s42520-021-00403-w

**Published:** 2021-12-17

**Authors:** Klaus Gestwa

**Affiliations:** Eberhard-Karls-Universität Tübingen, Tübingen, Deutschland

**Keywords:** Geschichtspolitik, Erinnerung, Putin, Osteuropa, Historiography, Memory, Politics, Putin, Eastern Europe

## Abstract

Der Beitrag trägt verschiedene geschichtspolitische Interventionen in Osteuropa zusammen und diskutiert vor allem am Beispiel der Geschichtspolitik des russischen Präsidenten Putin Versuche, durch historische Umdeutungen politische Ziele zu legitimieren und von gesellschaftlichen Krisen abzulenken. Zunächst werden die zivilgesellschaftlichen Ursprünge sowie Kommerzialisierung und Institutionalisierung des geschichtlichen Interesses in den post-sowjetischen Ländern skizziert. Anschließend analysiert der Beitrag am Beispiel des russisch-estnischen Denkmalstreits sowie einer Auseinandersetzung über die Interpretation des Zweiten Weltkriegs die Bedeutung von Geschichtspolitik als Mittel von Spaltung oder Versöhnung. Die geschichtspolitische Landschaft in Osteuropa wird als umkämpftes „Schlachtfeld der Erinnerungen“ charakterisiert.

## Abstract (en)

This article discusses attempts to legitimise real political goals through historical reinterpretations or to divert attention from political crises, using the example of Russian President Putin’s historical policy. After briefly outlining the origins of civil society as well as the commercialisation and institutionalisation of historical interest in the post-Soviet countries, the article analyses the examples of the Russian-Estonian monument dispute as well as a dispute over the interpretation of the Second World War between Russia, Ukraine and Lithuania. The historical-political situation in Eastern Europe is characterised as a “battlefield of memories”.

Runde Jahrestage bieten oft einen guten Anlass, um die Erinnerung an historische Wendepunkte auf ein neues Niveau zu heben. Ein „gesamtdeutsch-russisches Symbol der Erinnerungskultur“^1^ stellte die bewegende Rede des russischen Schriftstellers Daniil Granin dar, die er anlässlich der Gedenkstunde an die Opfer des Nationalsozialismus am 27. Januar 2014 vor dem Deutschen Bundestag hielt. Dieser Tag war doppelt gut gewählt, weil sich damals zum 70. Mal sowohl das Ende der Blockade Leningrads durch die Wehrmacht jährte als auch die Befreiung des Vernichtungslagers Auschwitz durch die Rote Armee. Mit eindringlichen Worten sprach der russische Ehrengast im Berliner Reichstagsgebäude davon, wie das deutsche Militär während der knapp 900-tägigen Belagerung Leningrads den Hunger als schreckliche Massenvernichtungswaffe einsetzte.^2^

Als Überlebender dieses nationalsozialistischen „Urbizids“^3^ blieb Granin bis zu seinem Tod 2017 eine anerkannte moralische Autorität. Das von ihm und Ales Adamovič herausgegebene „Blockadebuch“^4^ half, die ideologischen Überblendungen der ritualisierten sowjetischen Kriegserzählungen aufzubrechen und mit einer „vielstimmigen Gegengeschichte“ einer eindimensionalen Sicht auf die Vergangenheit entgegenzuwirken.^5^ Jenseits der offiziellen Historiografie mit ihrem heroischen Mythos um den „Großen Vaterländischen Krieg“ rückte Granin die individuelle Leiderfahrung und problematische Seiten der Weltkriegsgeschichte ins Licht. Unter anderem problematisierte er die zweifelhafte Rolle der Parteiführung bei der Verteidigung Leningrads, die sich in Zeiten des omnipräsenten Hungertods fürstlich bewirten ließ.^6^ In seiner Rolle als patriotischer Kriegsveteran, der als unerbittlicher Kämpfer gegen die Wehrmacht später seinen Frieden mit Deutschland schloss, ist Granin ein bewundernswerter Botschafter der erfolgreichen deutsch-russischen Versöhnungsarbeit.

## Wider den historischen Masochismus: Geschichte als kollektive Seelenwellness

Auch der russische Präsident Vladimir Putin hielt zu Beginn seiner ersten Amtszeit am 25. September 2001 eine viel beachtete Rede vor dem Deutschen Bundestag, den Großteil davon sogar in einem ausgezeichneten Deutsch. Seine spärlichen Hinweise auf positive Aspekte der deutsch-russischen Beziehungsgeschichte dienten vor allem dazu, sein Hauptanliegen zu unterstreichen. Vor dem Hintergrund der kurz zuvor am 11. September 2001 in den USA verübten Terroranschläge bedürfe es einer neuen globalen Sicherheitsarchitektur, in der Russland und seine Interessen angemessene Berücksichtigung finden müssten, um die gegenwärtigen globalen Herausforderungen zu meistern.^7^

Als in der Folgezeit die Sowjetnostalgie in Russland um sich griff, begann Putin, sich zunehmend für den politischen Nutzen der Geschichte zu interessieren. Dabei trat er zunächst weniger mit programmatischen Reden zur Geschichtspolitik in Erscheinung. Vielmehr griff er mit eingängigen Formulierungen die in der Gesellschaft verbreiteten historischen Sehnsüchte auf und bestärkte schon bestehende geschichtspolitische Konjunkturen.

Mit Gorbatschows *Glasnost* hatte seit 1987 die schmerzhafte Auseinandersetzung mit historischen Tabuthemen begonnen. Diese in der postkommunistischen Dekade fortgesetzte schonungslose Aufarbeitung erschütterte zunehmend das kollektive Selbstwertgefühl in Russland. Gegen Ende der 1990er Jahre fanden Stimmen in der politischen Öffentlichkeit zunehmend Gehör, die von einem unerträglich gewordenen historischen Masochismus sprachen und einen neuen Erinnerungscode einforderten, um der vermeintlichen Pädagogik der Scham und Selbsterniedrigung ein Ende zu setzen. Statt weiter in den offenen Wunden der Vergangenheit herum zu bohren, galt es, zum Zwecke kollektiver Seelenwellness wieder stärker die Errungenschaften der Sowjetgeschichte in Erinnerung zu rufen.^8^ Der Blick zurück sollte nicht mit Zorn, sondern mit Stolz erfüllt sein, um anstelle des kompromittierten Sozialismus fortan mit einem pathetischen Patriotismus eine positive Bindung der Russ_innen an ihren Staat zu schaffen. Diese positive Rückbesinnung wurde von den staatlichen Stellen vorangetrieben. Sie schlug sich im Jahr 2000 für alle hör- und sichtbar in der Wiedereinführung der alten sowjetischen Hymne (mit neuem Text) und in zahlreichen weiteren Rückgriffen auf die sowjetische Staatssymbolik nieder.^9^

Besondere Aufmerksamkeit erhielt das von Putin in seiner Rede zur Lage der Nation im April 2005 verkündete Statement, der Zerfall der Sowjetunion 1991 sei „die größte geopolitische Katastrophe“ des 20. Jahrhunderts gewesen.^10^ Während der Kremlchef damit auf den Punkt brachte, was viele russische Bürger_innen beschäftigte, griff im Ausland die Furcht um sich, Moskau könne zur Linderung neoimperialer Phantomschmerzen eine expansive Sammlungspolitik der Länder der ehemaligen Sowjetunion oder sogar des Ostblocks anstreben. Diese Ängste wuchsen, als Russland im März 2014 die ukrainische Halbinsel Krim völkerrechtswidrig annektierte und kurz darauf fünf postsowjetische Nachfolgestaaten das Gründungsabkommen der von Moskau dominierten Eurasischen Wirtschaftsunion unterschrieben.^11^

Geschichtspolitisch fiel Putin ferner mit lobenden Äußerungen zur Arbeit des sowjetischen Geheimdienstapparats auf, der viele stalinistische Gräueltaten exekutiert hatte und damit während der kritischen Aufarbeitung der sowjetischen Geschichte in den 1990er Jahren zum Inbegriff totalitärer Schreckensherrschaft geworden war. Der vom KGB-Spion zum Präsidenten aufgestiegene Putin leugnete die stalinistischen Gräueltaten zwar nicht. Aber er relativierte sie, indem er die Grausamkeiten der damaligen Machthaber als politische Notwendigkeit auslegte. Nur durch den brutal erzwungenen Gehorsam und seinen erbarmungslosen Industrialisierungsfeldzug habe Stalin mit seiner starken Führung dem Land den Weltkriegstriumph bringen können. Das müsse bei der historischen Bilanz des Stalinismus entsprechende Gewichtung finden. Ansonsten diene die „exzessive Dämonisierung Stalins“ – so Putins Deutung – nur dazu, „die Sowjetunion und Russland anzugreifen“.^12^ Formulierungen trugen dazu bei, dass das zwischen 2009 und 2012 von Politik und Öffentlichkeit in Russland geforderte „Programm zur Entstalinisierung“ scheiterte. Prostalinistische Gegenkampagnen erklärten, „Russophobie und Entstalinisierung sind zwei Seiten einer Medaille“. Den Entstalinisierer_innen wurde ungeniert vorgeworfen, sie „tanzen nach der Pfeife der Provokateure aus dem Westen“, wollten „Russland in einem uferlosen Minderwertigkeitskomplex ertränken“ und mit einer historischen „Hexenjagd“ einen „Bürgerkrieg“ lostreten.^13^ Zwar musste das von der Moskauer Staatsverwaltung 2010 gestartete Unterfangen gestoppt werden, anlässlich des 65. Jahrestags des Weltkriegstriumphs zahlreiche Werbeflächen und Stadtbusse mit den Bildnis Stalins zu versehen, um so an seinen „herausragenden Anteil an der Zerschlagung des Faschismus zu erinnern“.^14^ Dennoch setzte sich der Trend zur Rehabilitierung Stalins weiter fort. Heute gilt er einer Mehrheit der russischen Bevölkerung wieder als bedeutendste historische Figur Russlands und großartiger Staatsmann, nachdem er gegen Ende der 1980er Jahre in der sowjetischen Öffentlichkeit in tiefe Ungnade gefallen war.^15^

Ein anschauliches Beispiel für das Bemühen, die stalinistische Schreckensherrschaft zu verharmlosen, ist das Schicksal des von zivilgesellschaftlichen Kräften getragenen GULag-Museums Perm-36. Diese auf dem Gelände eines vormaligen Arbeitslagers liegende Gedenkstätte geriet nach 2012 unter starken politischen Druck. Die Museumsleitung wurde komplett ausgetauscht und die Trägerschaft neu geregelt. Infolgedessen kam es zu einem grundlegenden Wandel des ursprünglichen Ausstellungskonzepts. Die Häftlinge erschienen nun nicht mehr als Opfer einer totalitären Diktatur, sondern als „Kriminelle“ und „Volksverräter“, die mit ihrer Zwangsarbeit gleichfalls einen Beitrag zum Weltkriegstriumph geleistet hätten.^16^

Gegen diese zynische Sichtweise intervenierten russische sowie internationale Wissenschaftler_innen und Medien. Damit erreichten sie zumindest, dass neuerdings in etwas angemessenerer Weise an das Leid ehemaliger Lagerinsassen erinnert wird.^17^ Die aktuelle Kritik an der Ausstellung bezieht sich weniger auf die Verherrlichung des GULag denn auf historische Ungenauigkeiten und die mangelhafte Authentizität. Als die eigentlichen Probleme erscheinen heute vor allem das Fehlen einer durchgängigen Narration und die sich daraus ergebende „moralische Neutralität“. Das erzeugt den Eindruck, „dass der Gulag alles und nichts bedeutet hat […], dass das halt passiert ist, aber egal“.^18^

## Wider das Vergessen: Geschichtspolitische Spektakel und zivilgesellschaftliche Erinnerungsarbeit

Die heftigen Debatten um Perm-36 zeigen, dass sich die auf gesellschaftliche Bedürfnisse reagierende Geschichtspolitik des Kremls mit konkurrierenden Erinnerungskulturen konfrontiert sieht. Zwar sind Teile der russischen Geschichtswissenschaft auf einen patriotischen Kurs eingeschwenkt und haben sich von professionellen Standards verabschiedet. Dennoch dauern seriöse historische Spurensicherung und Aufarbeitung auch unter erschwerten politischen Arbeitsbedingungen ungebrochen an. Weiterhin gibt es in Russland engagierte, kritische Forscher_innen, die sich – nicht selten mit staatlichen Fördergeldern – erfolgreich in internationale Forschungsverbünde und Kooperationsvorhaben einbringen, um auf wichtigen akademischen sowie politischen Themenfeldern klare Positionen zu beziehen. So hat der Moskauer Verlag ROSSPEN eine vielbändige Stalinismus-Edition herausgegeben. Die hier veröffentlichten seriösen Werke, darunter auch zahlreiche in Russische übersetzte westliche Studien, waren schnell ausverkauft und wurden als (illegale) digitale Kopien aus dem Internet massenhaft heruntergeladen.^19^ Bestes Beispiel für eine kritische Auseinandersetzung mit der sowjetischen Geschichte ist Oleg Chlevnjuks in Russland mehrfach ausgezeichnete Stalin-Biografie, die in guter Kenntnis der Quellen weit verbreitete Geschichtsmythen dekonstruiert und Stalin als skrupellosen Gewaltherrscher darstellt, der mit dem Leben von Millionen Menschen Roulette spielte.^20^

Eine im Februar 2020 durchgeführte Umfrage unter russischen Historiker_innen ergab allerdings, dass die große Mehrheit von ihnen in ihrer akademischen Profession keine Kraft sieht, die das Geschichtsbewusstsein der Gesellschaft stark beeinflusst. Eine knappe Mehrheit der befragten Historiker_innen meinte, es gebe gegenwärtig einen staatlichen Auftrag für sie, historische Argumente zu liefern, um politische Aufgaben zu lösen. Tatsächlich legen Äußerungen des russischen Kulturministers Vladimir Medinskij nahe, dass Moskauer Regierungskreise Geschichte nicht als wissenschaftliche Disziplin, sondern vielmehr als ein Reservoir staatstragender Mythen verstanden wissen wollen. Mit dieser Sicht auf die Vergangenheit zeigen sich Historiker_innen aller Altersgruppen jedoch mehrheitlich unzufrieden und fordern, dass sich staatliche Behörden aus Geschichtsdiskussionen heraushalten und diese nicht manipulieren sollten.^21^

Weit mehr gesellschaftliche Breitenwirkung als die Geschichtswissenschaft erzielen russische Publizist_innen und Künstler_innen in Romanen und Memoiren, in Essays und Fernsehdokumentationen. Auch die Gruppe der Blogger_innen behandelt über digitale Medien problematische Aspekte der sowjetischen Geschichte auf anspruchsvolle und differenzierte Weise.^22^ Ihre Aktivitäten haben die russischen Behörden allerdings im Vorfeld der Duma-Wahlen im September 2021 noch einmal stark durch Zensurmaßnahmen eingeschränkt. Der massive Druck auf Internet-Provider, die Blockade von „GoogleDoc“ und die Sperrung kritischer Websites sowie Portale weisen, genauso wie die heftigen Cyberattacken auf zahlreiche Presseorgane, daraufhin, dass die russischen Behörden gewillt sind im „Informationskrieg“ gegen unliebsame Meinungen noch repressiver vorzugehen.

Besondere Aufmerksamkeit fand im April 2019 der russische Journalist und Videoblogger Jurij Dud’ (geboren 1986), als er seinen zweistündigen Film „Kolyma – Heimat unserer Angst“ online stellte. Der in Ostsibirien gelegene Fluss Kolyma gilt als Synonym für den stalinistischen GULag, weil sich hier einer der schlimmsten Lagerkomplexe befand. Zwischen 1931 und 1957 mussten in diesem entlegenen Vorhof der Hölle knapp eine Million Häftlinge Zwangsarbeit verrichten.^23^ Der Dokumentarfilm ist als Reisereportage konzipiert. Er lässt viele Betroffene und Historiker_innen zu Wort kommen, um ihre Erfahrungen mit der brutalen Lagergeschichte aufzuzeichnen und damit für die Nachwelt festzuhalten. Mit seiner historischen Aufklärungsarbeit richtet sich Dud’ besonders an die junge Zuschauerschaft, um deren Leerstelle im Geschichtsbild auszufüllen. Laut einer repräsentativen Umfrage aus dem Jahr 2018 hat knapp die Hälfte der 18- bis 24-Jährigen in Russland noch nie etwas von den stalinistischen Säuberungen gehört. Der Dokumentarfilm traf dank seiner auf die junge Zielgruppe ausgerichteten Darstellung und Diktion den Nerv der Zeit. Innerhalb des ersten Monats war er schon knapp 10 Millionen Mal angeklickt worden. Ein Jahr später überstiegen die Aufrufe die 20 Millionen-Marke. Mittlerweile verzeichnet „YouTube“ knapp 25 Millionen Klicks und über eine Million Likes. Damit gehört die historische Dokumentation über das eisige Schattenreich von Kolyma zu den erfolgreichsten russischen Videos der letzten Jahre.^24^

Mit seiner enormen Popularität löste der Film eine heftige Diskussion in den russischen Medien aus. Kritische Stimmen unterstellten Dud’ „fehlenden Patriotismus“ und warfen ihm vor, die Geschichte Russlands „in den Dreck ziehen zu wollen“. Das führe zu „Verwüstung in den Seelen von Millionen Russen“. Andere lobten die aufklärerische Leistung des Films, der mit seinem schonungslosen Blick in den mörderischen Abgrund des Stalinismus „all die faulige Sowjetnostalgie, all die masochistische Liebe zu Stalin-Stiefeln, das gewissenlose Jonglieren mit Orden toter Kriegsveteranen und das Posieren in Soldatenmänteln“ entzaubere.^25^

Die Erinnerung an die menschenverachtende Welt der Lager und der Zwangsarbeit hält auch das „Staatliche Museum der Geschichte des GULag“ aufrecht. Gegründet 2001, zog es 2015 in ein deutlich größeres Gebäude außerhalb der Moskauer Innenstadt um. Zusammen mit Archiven, Privatpersonen, Künstler_innen und Historiker_innen veranstaltet das Museum zahlreiche kulturelle Aktivitäten, bei den neben den Opfern auch die Täter des stalinistischen Terrors und damit die kritischen Seiten der sowjetischen Geschichte facettenreich, wenn auch nicht lückenlos thematisiert werden.^26^

Aufsehen erregt gleichsam die von Sergej Parchomenko, einem Moderator des Radiosenders *Echo Moskvy*, 2014 gestartete Bürgeraktion „Letzte Adresse“. Bei dieser Kampagne, die sich auf die Grundidee des deutschen Vorhabens „Stolpersteine“ bezieht, werden Gedenktafeln für die Opfer des stalinistischen Terrors an der Außenfront ihres letzten bekannten Wohnhauses angebracht.^27^

Gegen das Vergessen der stalinistischen Repressionen arbeitet zugleich das Projekt „Topografie des Terrors“. Dabei handelt es sich um einen interaktiven, digital verfügbaren Stadtplan, der Täter- sowie Opferorte verzeichnet und dazu wichtige Informationen vermittelt, um so das heutige Moskau und andere russische Städte mit einem Netz von alltäglichen Erinnerungsorten zu überziehen.^28^

Die Wachsamkeit der Zivilgesellschaft führte 2019 in der Uralmetropole Jekaterinburg dazu, dass ein besonders massiver Vorstoß staatlicher Behörden und Vertreter_innen der Sicherheitskräfte scheiterte, unbequeme Geschichte zu entsorgen. Dort sollte die auf dem Gelände eines stalinistischen Massengrabs errichtete Erinnerungsstätte den Schießständen eines zum Innenministerium gehörenden Sportvereins weichen. Dieser zynische Akt, an einem Ort früherer Massenerschießungen die Geheimpolizei wieder den Waffengebrauch trainieren zu lassen, löste monatelange Demonstrationen aufgebrachter Anwohner_innen aus. Diese verhinderten schließlich, dass auf den Überresten von 20.000 verscharrten Terroropfern Zielscheiben aufgestellt wurden.^29^

Für einen veritablen Skandal sorgte zuletzt noch einmal im Januar 2021 die Eröffnung des Imbisses „Stalin Döner“ im Norden Moskaus, dessen Personal in grünen Geheimdienstuniformen aus den 1930er Jahren bediente. Diese geschmacklose Marketing-Idee rief einen Sturm der Entrüstung hervor und führte zur Schließung der Dönerbude. Die Menschenrechtsorganisation „Memorial“ kritisierte, derartig deplatzierte und abstoßende Initiativen seien nur möglich, weil der russische Staat es bislang vermieden habe, sich von den Verbrechen unter Stalin in aller Klarheit abzugrenzen. Deshalb habe sich die Rechtfertigung des massenhaften Staatsterrors zu einer Normalität entwickelt. Dazu trage maßgeblich bei, dass stalinistische Begriffe wie „Volksfeinde“ und „ausländische Agenten“ längst wieder Teil des Sprachgebrauchs der russischen Politik, Verwaltung und Öffentlichkeit geworden seien.^30^

Als Reaktion auf diese zivilgesellschaftliche Kritik hatten Präsident Putin und der Patriarch der Russisch-Orthodoxen Kirche schon am 30. Oktober 2017 (nunmehr offizieller Gedenktag an die „Opfer der politischen Repressionen“) die „Mauer der Trauer“ eingeweiht. Dieses im Moskauer Stadtzentrum errichtete Denkmal mit weitreichendem Deutungsanspruch soll einerseits an die Opfer der Stalin-Ära erinnern, andererseits aber einen Schlussstrich unter die fortwährende Geschichtsdebatte ziehen. Das Denkmal bietet zwar ein hohes Identifikationspotential; doch regt sich vielerorts Kritik. Bemängelt werden vor allem die Verstaatlichung der Erinnerung und die Vernachlässigung der Täter_innen, sodass es zu keiner Thematisierung der Frage nach Schuld und Verantwortung kommt. Zudem gilt das Denkmal als Ausdruck politischer Scheinheiligkeit, weil in der Amtszeit Putins ungeniert politisch motivierte Gerichtsprozesse angestrengt würden, immer mehr politische Gefangene in Haft säßen und der Zugang zu politisch brisanten Archivbeständen zunehmend eingeschränkt werde.^31^

Schon 1988 hatte der sowjetische Historiker Michail Gefter angesichts der enormen Gegenwartspräsenz des Stalinismus plakativ postuliert: „Stalin ist erst gestern gestorben“.^32^ Die bis heute „verzerrte Trauer“ habe das postkommunistische Russland sodann immer mehr zu einem „Land der Unbeerdigten“ werden lassen. Die zahlreichen Opfer der stalinistischen Terrorherrschaft verfolgen als „Untote“ weiterhin die Nachgeborenen. Bei den Auseinandersetzungen mit der unverarbeiteten Gewaltgeschichte kehren traumatische Leiderfahrungen immer wieder aufs Neue auf die Bühne der Politik zurück, um dort neue, oftmals gespenstische Spektakel zur Aufführung zu bringen.^33^

## Institutionalisierung und Kommerzialisierung von Geschichte

Als sich bei den Polittechnokrat_innen des Kremls die Meinung durchsetzte, dass eine „gelenkte Demokratie“ ein intensiv betreutes historisches Gedenken benötige, gründeten sie 2009 die „Präsidentielle Kommission zur Verhinderung der Fälschung der Geschichte zum Schaden der Interessen Russlands“. Zwar verschwand diese Überwachungsagentur schon fünf Jahre später sang- und klanglos wieder von der politischen Bildfläche; aber ihre Gründung zeigte, dass die staatlich kontrollierte Institutionalisierung der Erinnerungskultur auf der politischen Agenda des Kremls stand.^34^ Es kam zu vermehrten administrativen und juristischen Schikanen gegen kritische Historiker_innen.^35^ Seit einem 2012 verabschiedeten Gesetz hat die Regierung die Möglichkeit, politisch tätige und international unterstützte zivilgesellschaftliche Organisationen als „ausländische Agenten“ zu registrieren und so zu diskreditieren (betroffen davon sind mittlerweile 160 Einrichtungen und Vereinigungen). Mit diesen administrativen Mitteln gehen die russischen Behörden erbittert und unnachgiebig gegen erinnerungskulturell aktive Organisationen wie „Memorial“ und das „Levada-Zentrum“ vor.^36^

Zugleich entstanden zahlreiche neue, explizit auf Geschichtsvermittlung ausgerichtete Gesellschaften und Vereine, die sich als NGOs bezeichnen, obwohl sie finanziell und personell eng mit Regierungsbehörden und staatsnahen Organisationen verbunden sind. Statt eine differenzierte Aufarbeitung voranzutreiben, ergehen sie sich meist in einer übermäßigen Glorifizierung der russischen und sowjetischen Vergangenheit, bei der problematische Aspekte verschwiegen, marginalisiert oder relativiert werden.^37^

Des Weiteren begann die Arbeit an verbindlichen, staatlich vorgegebenen historischen Bildungsstandards. Dafür hat sich besonders die von 2016 bis 2020 amtierende, als eifrige Hüterin patriotisch-religiöser Werte auftretende Bildungsministerin Ol’ga Vasil’eva eingesetzt.^38^ Bereits für Kindergärten gibt es mittlerweile Programme zur patriotischen Erziehung. Viel diskutiert werden seit einigen Jahren die Vorgaben für den Geschichtsunterricht und die neuen Geschichtslehrbücher.^39^ Zudem trägt die in Moskau und mittlerweile in weiteren 25 Städten organisierte Großausstellung „Russland – Meine Geschichte“ dazu bei, ein politisch gewünschtes Bild von der russischen Vergangenheit zu vermitteln. Diese mit modernen immersiv-multimedialen Mitteln gestaltete Exposition haben die Kurator_innen im Auftrag des russischen Kulturministeriums und der russisch-orthodoxen Kirche dank großzügiger finanzieller Förderung von Gazprom realisiert.^40^

Damit einhergegangen ist eine Kommerzialisierung durch eine neue „Geschichtsindustrie“.^41^ Mit staatlichen Geldern produzierte, patriotisch eingefärbte Historienfilme haben im Format von Hollywood-Blockbustern mit flachen Dialogen, platten Genreklischees, lauten Spezialeffekten und übertriebenem Pathos Geschichte auf die Leinwand gebracht. Im russischen Fernsehen sind Serien und Sendungen beliebt, die bei ihrem Anspruch, angeblich unbekannte historische Wahrheiten aufzudecken, vom allgemein gesicherten Forschungsstand stark abweichende Behauptungen präsentieren. Das Ziel dieser kostümierten Geschichtspolitik ist es meist, mit ihrer überzeichneten Emotionalisierung und Effekthascherei Russland und die Sowjetunion von historischer Verantwortung reinzuwaschen. Deshalb wird mittels vermeintlich spektakulärer neuer Funde die Schuld ausländischen Akteuren zugewiesen. Nicht selten erfahren damit überlieferte Verschwörungsfantasien eine Aktualisierung.^42^ Beim kinematografischen Bleichen der dunklen Seiten der russisch-sowjetischen Geschichte erscheinen die einfachen Menschen nur als reines Verschleißmaterial des großen Weltgeschehens, während die Staatsmacht sakralisiert und ihre in bestimmten Phasen besonders evidente autoritäre Härte relativiert wird.^43^

## Im Gleichschritt Marsch: Die Militarisierung von Geschichte

Eine besondere Geschichtslektion in Form eines patriotischen Vergnügens erteilt ferner der als „militärisches Disneyland“ bezeichnete, eine Autostunde westlich vom Moskau gelegene „Park Patriot“. An seiner Eröffnung im Juni 2015 nahm Präsident Putin genauso teil wie führende Vertreter des russischen Rockerklubs „Nachtwölfe“. Diese Vereinigung vertritt nationalistische, neostalinistische und christlich-orthodoxe Ansichten. Ihre Mitglieder bilden gern die Staffage, vor der sich der russische Präsident als cooler Biker in Szene setzt. Der vom russischen Verteidigungsministerium betriebene Park zeigt zahllose militärische Exponate und bietet einen verklärten Blick auf die bis heute fortdauernde russische Kriegsgeschichte (Teil der Ausstellung ist auch die russische Intervention im syrischen Bürgerkrieg). Die Geschichte Russlands erscheint hier als eine Abfolge großer militärischer Siege.

Darüber hinaus kam es im April 2017 zur Einweihung eines Nachbaus des deutschen Reichstagsgebäudes, um in Form eines *reenactment* die Schlacht um Berlin und den Sturm auf den Reichstag nachspielen zu können. Der blutige Weltkrieg verkommt so zu einem harmlosen Spektakel, um die Erinnerung daran zu kommerzialisieren und sie auf dem Markt der Ideologien und Identitäten zur Ware zu machen. Zusätzlich zu den interaktiven Wissens- und Erlebnisräumen im patriotischen Jugendbildungszentrum „Avantgarde“ dienen derartige Inszenierungen dazu, den überlieferten „Fanfarenheroismus“^44^ auf die nachwachsende Generation zu übertragen und Kritik an der sowjetischen Kriegsführung zu unterbinden.^45^

Ähnliche, von Putin geforderte „lebendige Formen des Patriotismus“ bilden auch die militärhistorischen Ferienlager und Festivals, die von der 2012 gegründeten „Russischen Gesellschaft für Militärgeschichte“ (RVIO) organisiert werden. Diese Veranstaltungen werden von mehreren Zehntausenden Kindern und Jugendlichen besucht, um ihnen durch das aktive Nacherleben von Geschichte (durch militärische Märsche und historische Touren sowie durch das Nachstellen von Schlachten und den Gebrauch alter Waffen) patriotischen Stolz auf die militärische Vergangenheit Russlands einzuflößen. Diese vorgegebenen und durchorchestrierten Aktivitäten, die kaum individuellen Gestaltungsraum bieten, dienen dazu, die Teilnehmenden daran zu erinnern, dass Russland „mit Blut getränkt und deshalb heilig ist“.^46^ Umfragen unter jungen russischen Menschen belegen, dass dieser geschichtspolitische Tatendrang Wirkung gezeigt und die Verbreitung eines pathetischen Triumphalismus befördert hat.^47^

Als ein besonderes Highlight des pompösen patriotisch-militaristischen „Park Patriot“ gilt die dort im Mai 2020 eingeweihte, sowohl dem Kriegstriumph 1945 als auch Christi Auferstehung gewidmete „Kathedrale der russischen Streitkräfte“. Sie veranschaulicht mittels der Sakralisierung des Weltkriegssiegs die Einheit von Regierung, Militär und Kirche. Ihre Errichtung wurde offiziell aus Spenden, faktisch aber mit Mitteln des Staates und russischer Rüstungsfirmen finanziert. Putin selbst stiftete aus seinem Privatvermögen die Hauptikone mit einem aufwändig gestalteten Christusbild.

Für heftigen Streit sorgte ein geplantes Wandmosaik, das Stalin und Putin zeigt, wie sie von einer Menschenmenge für den Sieg über Hitler-Deutschland 1945 und für die Annexion der Krim 2014 gefeiert werden. Während die russische Militärführung Stalin als siegreichen Kriegsherren längst wieder in ihr Pantheon aufgenommen hat, hagelte es Kritik vonseiten der Medien und russisch-orthodoxer Priester. Die Erinnerung daran, dass Stalin während der 1930er Jahre einen erbitterten Feldzug gegen die Religion führte und dabei über 90 Prozent des russisch-orthodoxen Klerus ermorden ließ, ist ungeachtet aller Beschönigungsversuche in vielen Kreisen keineswegs verblasst.^48^ Angesichts der großen Entrüstung über das fast fertige Wandmosaik kam dieses dann doch nicht in die Kathedrale, sondern landete in einem benachbarten Museum.^49^

## Unbequeme Fragen

Bezogen auf die Erinnerung an den „Großen Vaterländischen Krieg“ als das Allerheiligste der Sowjetgeschichte sprach schon der Schriftsteller Vasilij Grossmann (1905–1964) von einem „stummen Streit zwischen dem siegreichen Volk und dem siegreichen Staat“.^50^ Dieser Zwiespalt ist längst nicht mehr unausgesprochen, seit im Zuge einer forschen historischen Offenbarungslust unbequeme Fragen gestellt werden. Als einer der größten russischen Fernsehsender (NTV) 2012 den nach einer wahren Begebenheit gedrehten deutschen Anti-Kriegsfilm „Vier Tage im Mai“ (Regie: Achim von Borreis) ausstrahlen wollte, kam es – angestiftet durch prostalinistisch-patriotische Organisationen – zu einem Medienskandal. Der Film, der das in Russland verdrängte Problem der massenhaften Übergriffe der Roten Armee auf die deutsche Zivilbevölkerung (auch die Massenvergewaltigungen) thematisierte, musste auf heftige Proteste patriotischer Gruppen hin aus dem Programm genommen werden. Er ziele angeblich nur darauf, die Kriegsveteranen zu verunglimpfen und die Ehre der Roten Armee in den Schmutz zu ziehen.^51^ Für politischen Wirbel sorgte 2014 auch der unabhängige TV-Sender „Dožd’“, als seine Redakteur_innen die schon 1989 vom russischen Schriftsteller Viktor Astaf’ev aufgeworfene provokante Frage, ob die Verteidigung Leningrads sinnvoll gewesen sei, zum Gegenstand einer Internet-Umfrage machten.^52^

Einen landesweiten Sturm der Entrüstung lösten im Sommer 2015 ferner die vom „Zentralen Staatsarchiv Russlands“ veröffentlichten Akten aus, die einen der bekanntesten sowjetischen Heldenmythen aus dem Zweiten Weltkrieg – die *Panfilovcy* – als *fake history* enthüllten. Drei Zeitungsjournalisten hatten noch während der Kriegshandlungen die Legende erfunden und erfolgreich verbreitet, Generalmajor Ivan Panfilov und 28 Infanteristen hätten im November 1941 vor Moskau trotz zahlenmäßiger Unterlegenheit einem massiven deutschen Panzerangriff standgehalten und dabei ihr Leben gelassen. Obwohl die veröffentlichten Archivunterlagen zweifelsfrei zeigten, dass an dieser patriotischen Erzählung über Selbstaufopferung und Kriegsruhm nichts stimmt, richtete sich die Wut von Öffentlichkeit und Politik keineswegs gegen die längst verstorbenen Fälscher_innen und die unbeirrten Bewahrer_innen der stalinistischen Propagandalüge, sondern gegen die historischen Aufklärer_innen. Ihnen wurde der Vorwurf gemacht, mit der „unmoralischen Demaskierung und Entzauberung der Heldentat“ an den Stützen der nationalen Identität zu rütteln. Die Botschaft war eindeutig: Die historische Wahrheit habe hinter geschichtspolitisch bedeutsamen Mythen zurückzustehen – Verklärung statt Enthüllung.^53^

Neben diesen vehementen Debatten um das richtige kollektive Gedenken ergibt sich die in Russland zu beobachtende „zerrissene Erinnerung“^54^ vor allem aus dem Zusammenwirken der gesellschaftlichen Verankerung der Kriegserfahrung mit der übergeordneten politischen Bedeutung der Kriegserinnerung für den Staat. Mit seinen 27 Millionen Toten und seinen enormen Entbehrungen ließ der „Große Vaterländische Krieg“ kaum eine sowjetische Familie unberührt und verursachte damit tiefe Spuren im privaten Gedächtnis der Generationen. Das daraus resultierende selbstbestimmte individuelle Gedenken prägen Siegesstolz und Trauer genauso wie die Thematisierung glorreicher Helden- und blutiger Schandtaten. Zu einem Spezifikum der dynamischen russischen Erinnerungskultur hat sich darum in der Putin-Ära das Bestreben entwickelt, das individuelle Gedenken im politischen Gedächtnis aufgehen zu lassen und so gesellschaftliche Eigeninitiative zu vereinnahmen.^55^

## Die Verstaatlichung des individuellen Gedenkens: Das „Georgsband“ und das „unsterbliche Regiment“

Ein anschauliches Beispiel für die Verstaatlichungsstrategie privater und sozialer Erinnerungspraktiken stellt das „Georgsband“ dar. Angelehnt an das britische *remembrance poppy*, verteilte die staatliche Nachrichtenagentur RIA Novosti mit Unterstützung der Moskauer Stadtregierung und einem studentischen Sozialhilfswerk 2005 am 9. Mai erstmals 800.000 schwarz-orange gestreifte Stoffschleifen. Als Zeichen der Dankbarkeit für die Veteranen des „Großen Vaterländischen Kriegs“ wurden sie zu einem neuen Symbol des Gedenkens, das explizit Traditionen aus der zarischen Militärgeschichte aufgriff und mit der sowjetischen Weltkriegsgeschichte in einen Zusammenhang stellte. Innerhalb kurzer Zeit entwickelte sich die Bandkampagne zu einem gesellschaftlichen Selbstläufer. 2007 trug Präsident Putin während der Mai-Feierlichkeiten das Bändchen zum ersten Mal auf großer öffentlicher Bühne am Revers. Er erhob damit das Gedenkbändchen zu einem wichtigen, staatlich sanktionierten Identitätsmarker.

Während der russischen Massenproteste gegen Putin 2011/12, bei denen weiße Bänder den allgemeinen Unmut zum Ausdruck bringen sollten, diente das aus der Mottenkiste der zarischen Militärgeschichte entlehnte „Georgsband“ kremltreuen Gegendemonstrierenden als Bekenntnissymbol ihrer Loyalität gegenüber dem Putin-Regime. Nach dem Umbruch in der Ukraine im Frühjahr 2014 und mit dem Beginn der russischen Aggression nutzten es die separatistischen Kräfte auf der Krim und in der Ostukraine, um damit ihre Verbundenheit mit Russland sowie ihre militärische Kampfbereitschaft zum Ausdruck zu bringen. Die beiden auf der Krim gelegenen Städte Sevastopol und Simferopol lieferten sich sogar einen Wettstreit um das längste „Georgsband“.^56^ Seitdem ist aus dem russischen *remembrance poppy* ein omnipräsentes, politisch aufgeladenes Gedenkemblem geworden, das für ein selektives Geschichtsbild steht. In diesem werden historische Brüche wie Revolution und Bürgerkrieg sowie alle negativen Vergangenheitsaspekte ausgeblendet, um eine ununterbrochene russisch-sowjetische Siegestradition zu konstruieren und dabei die geschichtsprägende Kraft des Militärs zu betonen.^57^

Aus dem verbreiteten Bedürfnis, jenseits großer Inszenierungen und bekannter Rituale eine eigenständige Erinnerungspraxis zu entwickeln, entstand die Initiative „unsterbliches Regiment“. In der westsibirischen Großstadt Tomsk versammelten sich 2012 erstmals Menschen zu einem Gedenkmarsch, um Porträts ihrer Verwandten vor sich herzutragen, die am „Großen Vaterländischen Krieg“ teilgenommen hatten.^58^ Diese zivilgesellschaftliche Aktion traf vielerorts in Russland auf große Resonanz. In den sozialen Netzwerken teilten russische Familien Berichte über die Kriegserlebnisse ihrer Vorfahren, die in ihrer unzensierten Form auch immer wieder Erzählungen vermittelten, die nicht dem tradierten, heroischen Narrativ entsprachen. Ziel dieses privaten Gedenkens im öffentlichen Raum war es, zum einen die Zugehörigkeit zur Erinnerungsgemeinschaft zu demonstrieren, und zum anderen durch die verstärkte Kommunikation von familienbezogenen Kriegserfahrungen die Erinnerungskultur – im wahrsten Sinne des Wortes – zu beleben.

Die Gedenkmärsche des „unsterblichen Regiments“ erhielten nicht nur in russischen Städten schnell enormen Zulauf. Zu ähnlichen Aktionen kam es auch in sowjetischen Nachfolgestaaten sowie in Ländern mit zahlreichen russischsprachigen Zuwander_innen, vor allem in Deutschland, Österreich, Israel und in den USA. Dieser rasante Erfolg hielt den Kreml dazu an, die neue Gedenkpraxis als Mobilisierungsressource zu nutzen und mit der neuen Erinnerungsform die Verbundenheit von Regime und Bevölkerung zu demonstrieren. Putin selbst nahm 2015 am Aufmarsch des „unsterblichen Regiments“ in vorderster Front teil und verlieh diesem damit höchste politische Weihen. Zudem erschienen seine Erinnerungen an die Kriegserzählung seiner Eltern in der Zeitschrift „Russkij Pioner“, um mit dieser emotionalen Aufladung die Geschichtspolitik des Staates anschlussfähig für Formen individualisierten Gedenkens zu halten.

Organisiert werden die Gedenkmärsche mittlerweile von regionalen historisch-patriotischen Vereinigungen, die sich als zivilgesellschaftliche Initiativen ausgeben, aber eng mit staatlichen Behörden, Stiftungen und Unternehmen verbunden sind, um so einen Zugriff der Polittechnokrat_innen des Kremls auf die neue gesellschaftliche Erinnerungspraxis zu ermöglichen.^59^

Auf die staatliche Vereinnahmung der gesellschaftlichen Aktion „unsterbliches Regiment“ hin entstand bald die Initiative „unsterbliche Baracke“, um zugleich an das Schicksal sowjetischer Lagerinsass_innen und Zwangsarbeiter_innen zu erinnern. Ihre Protagonist_innen mahnen, dass nicht nur der Zweite Weltkrieg, sondern auch die stalinistische Lagerwelt lange Schatten bis in die russische Gegenwart wirft und deshalb als unbewältigtes Erbe ernst zu nehmen ist. Diese Kampagne findet bei weitem keinen vergleichbaren Zulauf. Sie verdeutlicht aber noch einmal, dass es in Russland soziale Kräfte gibt, die sich dem geschichtspolitischen Mainstreaming der Polittechnokrat_innen des Kremls hartnäckig widersetzen.^60^

## Patriotischer Jubel und historische Jubiläen: 2014 und 2017

Seit der Annexion der Krim ist Putin selbst verstärkt auf dem Schlachtfeld der Erinnerung mit geschichtspolitischen Salven in Erscheinung getreten. Er operiert auf dem Boden eines Halbwissens, um besser weglassen, beschönigen und umdeuten zu können. In seiner Rede anlässlich des „Beitritts“ der Krim zur Russischen Föderation gab der sichtbar stolze Präsident am 18. März 2014 einen Deutungsrahmen vor, um der russischen Einverleibung der ukrainischen Halbinsel historischen Sinn und politische Legitimität zu geben. Dafür prägte er das Narrativ vom „Triumph der historischen Gerechtigkeit“.^61^

Als wenige Monate später der Jahrestagsrummel um den Beginn des Ersten Weltkriegs ausbrach, brachte sich Putin aktiv darin ein, dem in Russland lange Zeit „vergessenen Krieg“ ein neues heroisches Narrativ zu geben. Er propagierte sein Konzept vom friedliebenden, aber von Feinden umzingelten Russland, das sich stets nur verteidigte und sich nicht scheute, für bedrohte Brüder und Schwestern in den Krieg zu ziehen. Wie vor 1914 sei Russland auch 100 Jahre später in Europa nicht gehört worden. Deshalb habe Moskau zum Schutz der auf der Krim lebenden russischen Menschen intervenieren und der Halbinsel als wichtigem Hort russischer Kultur den „Weg in die Heimat“ bereiten müssen.

In einer merkwürdigen geschichtspolitischen Volte erklärte Putin zudem, Russland sei der Sieg im Ersten Weltkrieg durch diejenigen gestohlen worden, die politische Zwietracht gesät und damit aus Machtgier die nationalen Interessen verraten hätten. Gerade in Krisen- und Kriegszeiten bedürfe Russland einer starken und geeinten Führung, der das Volk uneingeschränktes Vertrauen entgegenzubringen habe. Dieses ganz auf die Gegenwart bezogene Geschichtsbild diente 2014 offensichtlich dazu, innere und äußere Aggressionen sowie autoritäre Ent- und Geschlossenheit zu rechtfertigen.^62^

In derselben Weise deuteten Putin und sein Gefolge drei Jahre später die beiden Russischen Revolutionen des Jahres 1917. Diese wurden mit Erschütterung, Kontrollverlust und Chaos explizit negativ konnotiert. Die Februarrevolution galt sogar als „erste Farbenrevolution“, um liberale Gesellschaftsentwürfe als „unrussisch“ zu denunzieren und mit dieser historischen Chimäre den ukrainischen „Euro-Majdan“ als existenzielle Bedrohung für Russland zu diskreditieren.^63^ Dazu passt, dass schon 2004 der ehemalige Revolutionsfeiertag vom 7. November durch einen neuen, drei Tage vorverlagerten Gedenktag ersetzt wurde. Dieser wird mit dem Bezug auf die Befreiung Moskaus von polnisch-litauischen Truppen 1612 seitdem als „Tag der Einheit des Volkes“ begangen, um nationalpatriotische Harmonien an die Stelle des vormaligen revolutionären Klassenkampfgedröhnes treten zu lassen und so den Menschen jegliche Lust auf Protest zu nehmen. Staatsbürgerliche Pflicht sei nicht Widerstand, sondern vor allem Gehorsam gegenüber der Staatsführung. Russland – so die Botschaft des Kremls – habe die schmerzhafte Periode der Revolutionen, Aufstände und Massenstreiks längst hinter sich gelassen. Unter Putins Führung sei die gesellschaftliche Zwietracht der Eintracht von Staat und Bevölkerung gewichen, um so die Ruhigstellung unzufriedener Geister zu simulieren sowie ungelöste Gegenwartsprobleme zu kaschieren.^64^ Bei den vom Kreml zum „Verfertigen der Wahrheit“ angewandten „Technologien der Seele“ kommt der Geschichtspolitik die zentrale Bedeutung zu, durch eine heilsame „Cliotherapie“^65^ das Wohlbefinden von Staat und Gesellschaft sicher zu stellen.^66^

## Geschichtsnarrative als Wegbereiter der Versöhnung oder machtpolitische Waffen?

Die sich nach aktuellen politischen Konjunkturen richtenden Erinnerungsoffensiven des Kremls zielen nicht nur innenpolitisch darauf, ein unabhängiges historisches Bewusstsein gesellschaftlicher Gruppen zu unterbinden, um durch eine gesteuerte geschichtspolitische Mobilmachung die Unterstützung für das Putin-Regime zu vergrößern. Darüber hinaus werden Geschichtsnarrative zu machtpolitischen Zwecken als Waffe in der hybriden Kriegsführung gegen Nachbarstaaten eingesetzt.

Während nach dem Zerfall des Sowjetimperiums und des Ostblocks die Transformationsgesellschaften in Osteuropa begannen, eigene nationale Narrative zu entwickeln, um sich ihre spannungsreiche Geschichte auf neue Weise anzueignen, gab es gleichzeitig Formen grenzüberschreitender geschichtswissenschaftlicher und erinnerungskultureller Kooperationen. Ziel war es, lange Zeit verdrängte Themen gemeinsam aufzuarbeiten und damit erste wichtige Schritte in Richtung Versöhnung zu gehen. Die gemeinsamen russisch-polnischen, ukrainisch-polnischen sowie russisch-baltischen Kommissionen und Projekte führten zu einer sorgsamen Dokumentation und politisch-historischen Neubewertungen des Hitler-Stalin-Pakts, des Warschauer Aufstands, des NKWD-Massakers von Katyn und weiterer Kriegs- und Nachkriegsverbrechen.

Ein Höhepunkt dieser Aufarbeitungs- und Versöhnungsinitiativen sollten anlässlich des 70. Jahrestags der Massenerschießungen von Katyn die gemeinsamen russisch-polnischen Gedenkfeiern vor Ort im Grenzgebiet Russlands zu Belarus sein. Sie wurden aber überschattet vom Absturz der polnischen Regierungsmaschine am 10. April 2010. Dabei starben der polnische Staatspräsident Lech Kaczyński und 95 weitere Passagiere, darunter hochrangige Politiker_innen und Vertreter_innen der Opferangehörigen. In Polen bot diese Tragödie nationalkonservativen Kreisen die Gelegenheit, antirussische Verschwörungsfantasien zu verbreiten. Das führte zu neuen geschichts- und außenpolitischen Spannungen.^67^

## Baltischer Testlauf: Der russisch-estnische Denkmalstreit 2007

Zu einer ersten aufsehenerregenden geschichtspolitischen Attacke Russlands auf einen Nachbarstaat kam es 2007 in Estland, nachdem die baltischen Staaten drei Jahre zuvor der EU und der NATO beigetreten waren. Zwischen Moskau und Tallinn gab es bereits zuvor wegen ungeklärter Grenzfragen und der Lage der russischen Minderheit Streit. Das umstrittene Gedenken an estnische Soldaten, die im Zweiten Weltkrieg für die deutsche Wehrmacht gegen die Rote Armee gekämpft hatten, trübte die Beziehungen weiter. In dieser schon angespannten Situation eskalierte der Konflikt um die Demontage des 1947 von einem estnischen Bildhauer und Architekten entworfenen Denkmalkomplexes für die im Zweiten Weltkrieg bei der Befreiung Tallinns gefallenen Rotarmist_innen. Das sowjetische Ehrenmal im Stadtzentrum sollte der Verbreiterung einer Hauptverkehrsstraße weichen.

Als die estnische Regierung taktisch unklug im April 2007 kurz vor dem symbolträchtigen Feiertag am 9. Mai die Zerlegung des Denkmals anordnete, kam es – angeheizt durch russische Medien und eingereiste Provokateur_innen – in Tallinn zu heftigen Ausschreitungen. In diesem „estnisch-russischen Kulturkampf“^68^ um die Deutungshoheit über den Zweiten Weltkrieg nutzte der Kreml das voreilige und kompromisslose Handeln der estnischen Regierung, um mit einer massiven Propagandakampagne den baltischen Nachbarn einzuschüchtern. Zugleich überprüfte Moskau, ob sich mit geschichtspolitischen Mitteln Verwirrung stiften und ein Keil in die EU treiben lässt. Dahinter stand das starke Verlangen, Russland wieder stärker als agierende Großmacht in der Arena der internationalen Politik in Stellung zu bringen.

Die estnische Regierung zog aus der geschichtspolitischen Kontroverse Lehren. Entschieden trat sie verbreiteten Gerüchten entgegen, dass ihre Politik auf die Tilgung sowjetischer Erinnerungsorte ziele. Der demontierte, den sowjetischen Befreiern gewidmete Denkmalkomplex wurde nicht eingeschmolzen, sondern auf einem Militärfriedhof wiederaufgebaut, um dort eine angemessene Gedenkstätte zu schaffen. Seitdem behandeln estnische Behörden emotional aufgeladene Streitthemen mit mehr Bedacht. Das hat dazu geführt, dass sich die russischsprachige Minderheit nun weniger anfällig für aggressive Stimmungsmache aus Moskau zeigt. Die im Denkmalstreit 2007 deutlich werdende russische Einmischung in innerestnische Angelegenheiten hat zudem die internationale Solidarität mit Estland gestärkt und dessen Integration in die EU gefördert.^69^

## Das geschichtspolitische Ringen um den Zweiten Weltkrieg: Der Streit mit der Ukraine und mit Litauen seit 2014

Der baltische Testlauf 2007 zeigte dem Kreml, wie gut sich der Zweite Weltkrieg mit seinen immensen Opfern auf sowjetischer Seite als ultimative Propagandagrundlage für den außenpolitischen Diskurs der Einkreisung durch den Westen und die verschwörungsgläubige Vorstellung von der unerschütterlichen „Festung Russland“ nutzen lässt.^70^ Nach der Annexion der Krim und dem Beginn der bis heute fortdauernden Intervention im ostukrainischen Donbass startete Moskau 2014 eine geschichtspolitische Offensive zur Demütigung und Bloßstellung der Ukraine, um die historische Legitimität und territoriale Integrität des Nachbarstaats infrage zu stellen. Erinnerungskulturelle Konkurrenzen und Kontroversen eskalierten in einem regelrechten Geschichtskrieg.

In den von Moskau aus geschürten Desinformationskampagnen und Propagandaschlachten erklärte der Kreml die heroische Tradition des „Großen Vaterländischen Krieges“ zum historischen Eigentum der russischen Nation. Zugleich wiesen seine Polittechnokrat_innen immer wieder darauf hin, dass ukrainische nationalistische Kräfte während des Zweiten Weltkriegs im Windschatten der deutschen Invasion zahllose Gräueltaten gegen Juden und Polen begangen hätten. Unter dem Label „Geschichte und Gegenwart des ukrainischen Faschismus“ brandmarkte der Kreml die im Frühjahr 2014 siegreichen Majdan-Protestierenden in Kiew pauschal als Horde von „Nationalisten“ und „Faschisten“. Diese plumpe Stigmatisierung der Ukrainer_innen als „faschistisches Tätervolk“ zielte einerseits darauf, die Ukraine mit geschichtspolitischen Mitteln international zu diskreditieren, um das Land zu einer einfachen Beute der russischen Aggression zu machen. Andererseits diente die seit 2014 exzessive, unter Zuhilfenahme sowjetischer Propagandaklischees betriebene Russifizierung des Weltkriegstriumphs und die maßlose Ukrainisierung der NS-Kollaboration dazu, mit dem überlieferten Mythos vom makellosen sowjetischen Antifaschismus die russische Intervention in der Ukraine als Fortsetzung des Kampfes gegen die NS-Barbarei präsentieren und damit legitimieren zu können.^71^

Im Sommer 2021 radikalisierte sich das Ukraine-Bild des Kremls noch einmal, als Putin die Geschichte bemühte, um eine Drohkulisse aufzubauen. Als der ukrainische Präsident Wolodymyr Selenskyj am 12. Juli 2021 auf Staatsbesuch in Berlin war und das russische Militär zuvor große Truppenverbände unweit der Grenze zur Ukraine zusammengezogen hatte, veröffentlichte der russische Präsident auf der Website des Kremls einen 5.000 Worte langen Grundsatzartikel zur „historischen Einheit von Russen und Ukrainern“. Dieser Text, der unverzüglich Pflichtlektüre der russischen Streitkräfte wurde, schlug einen großen historischen Bogen von dem mittelalterlichen Staatengebilde der Kiewer Rus bis in die Gegenwart, um unter Missachtung geschichtswissenschaftlicher Methoden und Kategorien zu belegen, dass „Russen, Ukrainer und Belarussen“ Teil einer „großen russischen Nation, eines dreieinigen Volkes“ seien, das durch den Zerfall der Sowjetunion eine grobe, künstliche Trennung erlitten habe. Zudem ließ Putin verlauten, Russland sei bei der 1922 durch die erste Verfassung des sowjetischen Unionsstaat erfolgten Gründung einer ukrainischen sozialistischen Teilrepublik zahlreicher Siedlungsgebiete und Menschen „beraubt“ worden.^72^

In seiner geopolitischen, ahistorischen und verschwörungstheoretischen Denkweise, deren Widersprüche, Falschbehauptungen und Manipulationen sofort in den sozialen Medien aufgedeckt wurden, dämonisierte Putin den „Euro-Majdan“ 2014 zum Höhepunkt eines uralten westlichen Plans, mit einer unter „externer Verwaltung“ stehenden, zu einem Vasallenstaat degradierten Ukraine ein „Anti-Russland“ zu schaffen. Mittels dieser bizarren Geschichtsklitterung werden in der neuen Moskauer Ukraine-Doktrin nicht nur die Legitimität und Souveränität der Ukraine in Frage gestellt, sondern auch Gebietsansprüche erhoben und der Welt noch einmal klar gemacht, dass der Kreml nur einen ukrainischen Nachbarstaat akzeptieren werde, der bereit ist, sich seinem hegemonialen Willen zu fügen.^73^

Auch das Beispiel Litauen zeigt, wie Russland auf dem „Schlachtfeld der Erinnerungen“^74^ in letzter Zeit geschichtspolitische Waffen einsetzt. In Litauen findet seit einigen Jahren eine öffentliche Debatte um den Zweiten Weltkrieg statt, darunter auch um die litauische Beteiligung an NS-Verbrechen wie dem Holocaust. Diese engagierte, aber auch schmerzhafte Aufklärungsarbeit litauischer Forscher_innen löste zunächst eine innergesellschaftliche Diskussion aus, wie sich eine integrierte litauisch-jüdische Geschichte jenseits aller Opferkonkurrenzen schreiben lässt. Im Schatten der hybriden Kriegsführung Moskaus gegenüber der Ukraine häuften sich dann russischsprachige Texte, die Litauen wegen der Verwicklung eines Teils seiner Bevölkerung in den NS-Vernichtungskrieg pauschal eine bis heute andauernde Anfälligkeit für den Faschismus unterstellten. Das Ringen Litauens um eine angemessene Holocausterinnerung erfuhr durch diese russische Einmischung und Verunglimpfung eine massive Politisierung. Der umkämpften Weltkriegsgeschichte kam als wichtiges Politikfeld zunehmende Aufmerksamkeit zu. Das schlug sich in einer wachsenden Zahl der Akteur_innen und Initiativen nieder, die seitdem neben ihren internen Auseinandersetzungen auch einen geschichtspolitischen Abwehrkampf gegen den Kreml auszufechten haben.^75^

Jenseits des rechten und linken Rands des politischen Spektrums^76^ entfaltete das geschichtspolitische Sperrfeuer aus Moskau auch in seriösen Teilen der westlichen Öffentlichkeit und Politik Wirkung. Bestes Beispiel dafür ist der Aufruf „Nie wieder Krieg in Europa? Nicht in unserem Namen!“ von „60 prominenten Persönlichkeiten aus Politik, Wirtschaft und Kultur“, darunter Roman Herzog, Gerhard Schröder, Antje Vollmer, Klaus Maria Brandauer und Wim Wenders. Es hieß im Aufruf, Russland gehöre seit dem Wiener Kongress 1814 „zu den anerkannten Gestaltungsmächten Europas“. Alle kriegerischen Vorstöße, die Machtposition Russlands in Europa zu verändern, seien „blutig gescheitert“, so wie zuletzt 1941 das „größenwahnsinnige Hitler-Deutschland“, das mit einem Vernichtungskrieg versuchte, „Russland zu unterwerfen“.^77^ Hinter solchen Äußerungen schimmert die 1939 von Carl Schmitt veröffentlichte Schrift „Völkerrechtliche Großraumordnung mit Interventionsverbot für raumfremde Mächte“ durch, auf die sich gerade auch kremlfreundliche rechtspopulistische Parteien vielerorts in Europa beziehen.^78^

Die Protagonist_innen des Aufrufs erinnerten an die deutsche Verantwortung für eine Friedenspolitik gegenüber Moskau, die sich allein schon aus dem hohen Blutzoll von 27 Millionen Kriegstoten ergebe, den die Sowjetunion für den Weltkriegstriumph hatte erbringen müssen. Zugleich war in großen Teilen der deutschen Öffentlichkeit eine überzogene Aufmerksamkeit für rechtsradikale Kreise in der Ukraine zu beobachten. Mit festem Blick auf Moskau reagierte die Berichterstattung damit offensichtlich auf den von russischen Geschichtskrieger_innen vermittelten irrigen Eindruck, die Mehrheit der ukrainischen Bevölkerung sei mit Nazideutschland im Bunde gewesen und fühle sich bis heute ihrem antisemitisch-nationalistischen Erbe verpflichtet.^79^

Nur sechs Tage nach dem Aufruf „Nie wieder Krieg in Europa?“ erschien ein Gegen-Aufruf. Darin bezogen Osteuropaexpert_innen unter der Überschrift „Friedenssicherung statt Expansionsbelohnung“ politisch Position, um mit ihrer Fachexpertise und der Mahnung „Fakten statt Pathos“ der dem Moskauer Sprachduktus aufsitzenden Politik- und Kulturprominenz deren historische Blindheit zu verdeutlichen.^80^ Wer an die aus dem nationalsozialistischen Vernichtungskrieg erwachsende historische Verantwortung erinnert, sollte beachten, dass die Ukraine neben Belarus besonders unter der Terrorherrschaft der NS-Besatzer_innen gelitten hatte, wie Timothy Snyder in seinem vielbeachteten Buch „Bloodlands“ eindrücklich darstellt.^81^

Knapp ein Drittel aller sowjetischen Kriegstoten hatten auf dem verwüsteten Territorium der Ukraine gelebt. 1,5 Millionen ukrainische Jüd_innen verloren durch den Holocaust ihr Leben. Die große Mehrheit der sowjetischen Zwangsarbeiter_innen, die aus ihrer Heimat ins Deutsche Reich verschleppt worden waren, stammte aus der Ukraine. Den Hunderttausenden ukrainischen Kollaborateur_innen standen drei Millionen ukrainische Rotarmist_innen gegenüber, die in den Reihen der sowjetischen Streitkräfte bei der Befreiung Europas fielen. Zudem ist die Kollaboration eine historische Bürde, an der alle vom nationalsozialistischen Deutschland unterjochten Nationen in Ost und West schwer zu tragen haben, darunter auch Russland. Das schmerzhafte, vielfach noch nicht umfassend aufgearbeitete Thema der NS-Kollaboration eignet sich nicht als Diffamierungsinstrument, um aus Nachbarstaaten historische Übeltäter zu machen und eigene Vorherrschaftsansprüche zu legitimieren.^82^

Die Ukraine selbst verhielt sich im Geschichtskrieg mit dem mächtigen Nachbarn Russland keineswegs passiv, sondern drehte ihrerseits an der Eskalationsspirale. Ziel war es das Leid der Ukraine während des Zweiten Weltkriegs endlich in der europäischen Erinnerungskultur zu verankern und die grausamen Folgen sowohl der nationalsozialistischen als auch der stalinistischen Terrorherrschaft zu thematisieren. Einen Sturm der Entrüstung gab es in Russland, als die Sängerin Jamala beim „Eurovision Song Contest“ 2016 den Sieg mit ihrem Lied „1944“ errang. Darin thematisierte sie die von Stalin verfügte Deportation der Krimtataren und trug damit zur Politisierung des populären Musikwettbewerbs bei.^83^

Ein Jahr zuvor hatte die ukrainische Regierung im Hauruck-Verfahren die umstrittenen „Dekommunisierungsgesetze“ zur angeblichen „Wiederherstellung der nationalen Erinnerung“ erlassen. Die von oben verordnete ethnonationale Meistererzählung schrieb der Geschichte der Sowjetukraine das Label einer kolonialen Fremdherrschaft zu. Die neuen Geschichtsgesetze verboten generell alle kommunistischen Symbole, legitimierten die bis dahin schon vollzogenen Denkmalstürze und regten weitere vorschnelle Umbenennungen an, die bei weitem nicht überall auf Verständnis stießen. Die staatliche Verordnung trat an die Stelle einer als Dialog mit gesellschaftlichen Gruppen organisierten Aufarbeitung. Dieses Vorgehen brüskierte viele Ukrainer_innen, die sich statt einer säuberungsähnlichen Entsowjetisierung des öffentlichen Raums eine breite, unvoreingenommene Diskussion über die quälende Vergangenheit mit all ihren Komplexitäten und Ambivalenzen gewünscht hätten.

Mit dem proklamierten „Abschied vom totalitären Erbe“ sind zwar auch Symbole strikt untersagt, die sich direkt auf den deutschen Nationalsozialismus beziehen, dafür aber nicht die in der Ukraine verbreiteten völkisch-rechtsextremen Embleme wie Wolfsangel und Schwarze Sonne. Zusammen mit der umstrittenen Heldenverehrung der ukrainischen Nationalbewegung, die im Zweiten Weltkrieg zeitweise mit der Wehrmacht und der SS kollaboriert hatte, brachte das der Ukraine den Vorwurf einer akuten Sehschwäche auf dem rechten Auge ein und befeuerte damit den von Moskau geschürten „antifaschistischen“ Diskurs.^84^

## Geschichte als Gloria der EU und Putins Replik

Ein abermaliges historisches Säbelrasseln – dieses Mal zwischen Russland und Polen – kündigte sich im Spätsommer 2019 an. Es nahm seinen Anfang mit dem am 19. September 2019 vom Europäischen Parlament angenommenen Entschließungsantrag zur „Bedeutung der Erinnerung an die europäische Vergangenheit für die Zukunft Europas“.^85^ Diesen hatten Polen und die drei baltischen Staaten vorangebracht, um ihrem zentralen geschichtspolitischen Anliegen mehr Nachdruck zu verleihen, endlich der ostmitteleuropäischen Erfahrung mit den gleichzeitigen Massenverbrechen des Nationalsozialismus und Stalinismus im gesamteuropäischen Gedächtnis mehr Bedeutung zu geben.^86^ Ziel war es, den Hitler-Stalin-Pakt als Ausgangspunkt des Zweiten Weltkriegs in den erinnerungskulturellen Fokus zu rücken. Durch die damit einhergehende Verschiebung der Koordinaten des europäischen Gedächtnisses jenseits aller nationalen und historischen Diversität sollte eine unifizierende EU-Erinnerungskultur hergestellt werden.^87^

Trefflich streiten lässt sich über die damit verbundene Vorannahme, die EU könne als europäisches Projekt im 21. Jahrhundert nur gelingen, wenn von Brüssel die Durchsetzung eines allen Mitgliedsstaaten politisch genehmen Kollektivgedächtnisses verfügt werde, um mit geschichtspolitischem Einheitskitt auseinanderdriftende Kräfte zusammenzuhalten. Die Erinnerungskultur mutiert damit von einem offenen Selbstverständigungs- und Aushandlungsprozess zu einem in sich geschlossenen, supranationalen Dekret.^88^ Neben dieser bedenklichen Instrumentalisierung der Geschichte als Gloria der EU verweisen Kritiker_innen auf die Gefahr, dass die Thematisierung der Verstrickung von Nationalsozialismus und Stalinismus die Totalitarismustheorie aus dem Kalten Krieg neu beleben könne. Die damit konstatierte verbrecherische Gleichrangigkeit von Faschismus und Kommunismus könne einem Geschichtsrevisionismus den Weg bereiten. Allerdings hat die historische Forschung in den letzten Jahren demonstriert, dass die vergleichende Zusammenschau von Nationalsozialismus und Stalinismus keineswegs eine Gleichsetzung impliziert. Ungeachtet aller Verflechtungen und Analogien fallen stets auch Abgrenzungen und Differenzen ins Auge, um damit den Forschungsstand nicht zu revidieren, sondern besser zu profilieren.^89^ Die Erkenntnis, dass viele Menschen in den Weltkriegs- und Nachkriegsjahren sowohl Täter als auch Opfer zweier Diktaturen waren, schärft den Blick für die Tragik der Umstände, in denen die Menschen ihr (Über‑)Leben einzurichten hatten und sich dabei in fatale Handlungskonstellationen verstrickten. Diese problematischen Erfahrungen lassen sich weder mit verklärenden Helden- sowie vereinfachender Leid- und Triumphgeschichten noch mit einem Gut-Böse-Raster fassen.^90^

Während der EU-Entschließungsantrag weitgehend unterhalb des Aufmerksamkeitsradars der deutschen Öffentlichkeit und Politik blieb, sah der Kreml darin einen geschichtspolitischen Fehdehandschuh. Seine Polittechnokrat_innen gingen aber geflissentlich darüber hinweg, dass Moskau an der offen aufflammenden Konfrontation nicht ganz unbeteiligt war. Am 22. und 23. August 2019 hatten Sergej Naryškin als Vorsitzender der „Russländischen Historischen Gesellschaft“ und Vladimir Medinskij als russischer Kulturminister den Hitler-Stalin-Pakt als eine „ausweglose Entscheidung“ und sogar als „diplomatischen Triumph“ Moskaus gefeiert. Dass im Zuge von Gorbatschows *Glasnost* die Moskauer Politik 1989 das Abkommen und die Zusammenarbeit der beiden Diktatoren entschieden verurteilt hatte, galt nun wieder als vorschnelle und „hysterische Verteufelung“.^91^

Auf diese Äußerungen hin lud Polens Staatsführung Putin nicht zur Gedenkfeier zum 80. Jahrestag des Beginns des Zweiten Weltkriegs nach Warschau ein und provozierte mit diesem Affront in Russland eine Welle antipolnischer Stimmungen. Das spielte den Befürworter_innen einer aggressiven Geschichtspolitik in die Hände, die aufgebracht darüber waren, dass die stärkere Akzentuierung des Hitler-Stalin-Pakts als Wegbereiter des Weltkriegs die Sowjetunion weniger als Opfer des nationalsozialistischen Vernichtungskriegs und Befreier Europas, sondern mehr als Komplize Hitlers und als totalitären Unheilbringer ins historische Rampenlicht rückte. Damit stand der Weltkriegstriumph als zweiter Gründungsmythos der Sowjetunion unmittelbar infrage. Das rüttelte an den identitätspolitischen Grundfesten des Putin-Regimes und widersprach dem historischen Empfinden der russischen Bevölkerungsmehrheit.

Im Dezember 2019 holte Putin zum geschichtspolitischen Gegenschlag aus, um Polen als zuvor ausgemachten Hauptgegner so schmerzhaft wie möglich zu treffen. Innerhalb von zwei Wochen thematisierte Putin zu öffentlichen Anlässen sechsmal kritisch den EU-Entschließungsantrag vom 19. September. Auf einem Treffen mit seinen Amtskollegen sowjetischer Nachfolgestaaten verstieg er sich sogar dazu, den verdutzten Staatschefs eine einstündige Geschichtsvorlesung zu halten und dazu aufzurufen, mittels einer gemeinsamen Historikerkommission für den postsowjetischen Raum ein einheitliches Geschichtsbild zu schaffen.^92^ Einige Tage später kam es dann zum Skandal, als Putin bei einer Sitzung des Verteidigungsministeriums Józef Lipski, den von 1933 bis 1939 amtierenden polnischen Botschafter in Berlin, mit Bezug auf dessen damalige diskriminierende Äußerungen als „Drecksack“ und „antisemitisches Schwein“ bezeichnete.^93^ Mit diesen Pöbeleien provozierte er Polen, dessen konservative Regierung 2018 ein umstrittenes Gesetz verabschiedet hatte, das untersagte, dem polnischen Staat oder Volk die Verantwortung oder eine Mitverantwortung für Verbrechen des nationalsozialistischen Besatzungsregimes zuzuschreiben.^94^ Als daraufhin der polnische Präsident Duda im Unterschied zum russischen Präsidenten auf dem 5. *World Holocaust Forum* in *Yad Vashem* im Januar 2020 keine Redezeit erhielt, sagte er seine Teilnahme ab. Putin hingegen nutzte seinen großen Auftritt vor internationalen Staatsgästen (darunter Bundespräsident Frank-Walter Steinmeier), um ungeniert seine Vorwürfe gegen Polen zu wiederholen und damit für einen Eklat zu sorgen.^95^

## Der Geschichtstraktat vom Juni 2020: Putins Interpretation des Zweiten Weltkriegs

Während des Jahres 2020 bestimmte zunächst die umstrittene Verfassungsreform das politische Geschehen in Russland. Sie diente vor allem dazu, Putin weitere Amtszeiten als Präsident zu ermöglichen. Ein Referendum sollte die neue Verfassung demokratisch legitimieren und unmittelbar nach Ende der Feierlichkeiten zum 75. Jahrestag des Weltkriegsendes stattfinden. Die Corona-Pandemie ließ den ausgeklügelten Terminplan des Kremls aber platzen und schuf mit ihrer akuten Bedrohungslage eine grundlegend veränderte gesellschaftliche Stimmung.^96^ Die geplante Militärparade fand daraufhin am 24. Juni und das Referendum eine Woche später statt.

Im Vorfeld dieser miteinander verbundenen politischen Ereignisse führte Putin persönlich einen Frontalangriff auf dem europäischen Schlachtfeld der Erinnerungen an, um die Geschichte des Zweiten Weltkriegs gegen die vermeintlichen heimtückischen Umschreibpläne des Westens offensiv zu verteidigen. Dies fand seinen Höhepunkt in der Publikation eines ausführlichen Grundsatzartikels Putins. Bei diesem besonderen Akt der Geschichtspolitik legte der russische Präsident seine Interpretation der Weltkriegsgeschichte dar und setzte weitere antipolnische Akzente. Sein Beitrag erschien zunächst am 18. Juni 2020 auf Englisch unter dem Titel „75th Anniversary of the Great Victory: Shared Responsibility to History and our Future“ auf der Website des konservativen US-Fachmagazins „The National Interest“ und am folgenden Tag auf Russisch auf der Moskauer Regierungsplattform *kremlin.ru* sowie in der „Rossijskaja Gazeta“, dem Amtsblatt der Regierung.^97^

Kurz darauf veröffentlichte die russische Botschaft in Berlin die russische Originalversion und eine deutsche Übersetzung auf ihrer Website.^98^ Wenige Tage später erhielten Deutschlands Osteuropa-Forscher_innen eine Mail von der Pressestelle der Botschaft. Darin wurde den Fachleuten allen Ernstes vorgeschlagen, „den Artikel von Wladimir Putin künftig bei der Vorbereitung von historischen Beiträgen zu nutzen“. Das löste ein lautes Rauschen im deutschen Blätterwald aus; zahlreiche angeschriebene Expert_innen machten sich die Mühe, Putins Darstellung als eine neue geschichtspolitische Volte des Kremls kritisch auseinanderzunehmen und die damit verbundenen politischen Absichten offenzulegen.^99^

Im Zentrum des langatmigen Traktats zum Zweiten Weltkrieg steht Putins eigenwillige Interpretation des Hitler-Stalin-Pakts. Dabei greift Putin ausgewählte, hinlänglich bekannte Fakten auf, um diese bei seinem akuten Rückfall in die ideologisierte Geschichtsschreibung für eine forsche, durch übermäßige Einseitigkeiten, plakative Unwahrheiten und subtile Botschaften geprägte Geschichtsklitterung zu nutzen. Vor allem versucht Putin zu kaschieren, dass Hitler das Bündnis mit Stalin dringend brauchte, um beim Überfall auf Polen einen drohenden Zweifrontenkrieg zu vermeiden und aus der Sowjetunion unbedingt benötigte Öl- und Agrarimporte zu erhalten. In trickreichen Verhandlungen mit Reichsaußenminister Joachim Ribbentrop konnte die sowjetische Seite eine für sie günstige „Beutepartnerschaft“ erreichen.^100^ Das berühmte Geheimprotokoll legte die Aufteilung Polens und Ostmitteleuropas durch neue Grenzverläufe fest, die der Sowjetunion durch eine Westausdehnung erhebliche territoriale Gewinne erbrachten, begleitet von massiver militärischer Gewalt und staatsterroristischen Exzessen im Zuge der Annexion.^101^

Mit der vollmundigen Deklaration des „Schutzes der historischen Wahrheit“ negiert Putin anders als die vormaligen sowjetischen Kremlchefs das Geheimprotokoll nicht mehr; aber er relativiert Stalins Rolle, indem er die Schuld an diesem Teufelspakt „europäisiert“ und vor allem „polonisiert“. Nachdem, so Putins Lesart, die europäischen Demokratien seit 1933 immer wieder versucht hatten, mit der nationalsozialistischen Diktatur politisch ins Geschäft zu kommen, hätte Stalin nicht abseits stehen können. Durch diese Umstände wäre er gezwungen gewesen, einen temporären Ausgleich mit Hitler zu finden, um Zeit für die dringend benötige Aufrüstung der sowjetischen Streitkräfte zu gewinnen.^102^ Putin verherrlicht Stalin keineswegs und spricht seine Terrorherrschaft im Inneren an; aber er betont, dass Stalin außenpolitisch umsichtig agiert habe, um letztlich sein Land und ganz Europa vor dem Nationalsozialismus retten zu können.

Als wichtiges Entlastungsargument für Stalins zeitweilige Allianz mit Hitler führt Putin – wenig überraschend – das Münchener Abkommen von 1938 an. Statt mit der Sowjetunion ein Bündnis einzugehen, hätten Großbritannien, Frankreich und Polen auf dem Altar der *Appeasement*-Politik die Tschechoslowakei geopfert. Der Kremlchef wirft den westlichen Historiker_innen und Politiker_innen vor, die Bedeutung des Münchener Abkommen als Auslöser des Zweiten Weltkriegs zu verschweigen. Damit verkennt er bewusst, dass dieses verhängnisvolle Vertragswerk allseits als misslungener diplomatischer Versuch in der europäischen Geschichte gilt und *Appeasement* längst zu einem Kampfbegriff geworden ist, um eine entgegenkommende Politik gegenüber machtgierigen Autokrat_innen zu brandmarken.^103^

Den Hinweis auf das Münchener Abkommen missbraucht Putin zugleich für eine Polemik gegenüber Warschau. Ebenso wie Ungarn habe auch Polen vom „Münchener Komplott“ profitiert, weil es nach der militärischen Besetzung des Sudetenlands durch Deutschland die tschechoslowakischen Teile des Teschener Olsa-Gebiets okkupierte und damit den Grenzkonflikt um das kleine Territorium in Ostschlesien zunächst für sich entscheiden konnte. Die diskreditierende Präsentation Polens als Besatzungsmacht an der Seite Hitlers überzieht jedoch und wirkt als leicht durchschaubares Manöver, um davon abzulenken, wie die Sowjetunion nach dem Hitler-Stalin-Pakt daran mitwirkte, Polen zu zerschlagen. So verliert Putin kein Wort darüber, dass Stalin und seine Vertrauten Polen in revanchistischer Weise als „hässlichen Bastard des Versailler Vertrages“ bezeichnet und darauf gehofft hatten, die nach dem Polnisch-Sowjetischen Krieg im Vertrag von Riga 1921 abgetretenen Gebiete wiederzuerlangen.^104^

Neben dem Münchener Abkommen sieht Putin im gleichfalls ohne Beteiligung Moskaus ausgehandelten Vertrag von Versailles ein „Symbol tiefer Ungerechtigkeit“ und damit einen wichtigen Wegbereiter des Zweiten Weltkriegs. Die 1919 geschaffene „Versailler Weltordnung“ beschwor – so Putin – mit ihren „willkürlich gestalteten Grenzen“ gegenseitige Ansprüche herauf, „die sich in Zeitminen verwandelten“. Das „Erbe von Versailles“ bildete gerade in Deutschland mit seiner „nationalen Demütigung […] den Nährboden für radikale und revanchistische Stimmungen“, sodass es den Nazis gelang das „deutsche Volk in einen neuen Krieg“ zu führen.

Bei seinem Schlenker zurück ins Jahr 1919 nutzte Putin gezielt den heute in der aktuellen politisch-publizistischen Rhetorik des Kremls vielbemühten Schlüsselbegriff der „Demütigung“. Er impliziert den Vorwurf, dass die westlichen Großmächte seit langem die internationalen Beziehungen und Grenzen allein nach ihren Wünschen gestalten, ohne auf die Interessen anderer Staaten Rücksicht zu nehmen.^105^ Deshalb komme es ständig zu „nationalen Demütigungen“, die in den davon betroffenen Gesellschaften Prozesse politischer Radikalisierung sowie aggressive Gegenschläge auslösen würden. Das Bild des durch den Vertrag von Versailles entwürdigten Deutschlands setzte Putin damit als Projektionsfläche für den heute in Russland populären „Erniedrigungsdiskurs“. Dieser wiederum dient dazu, die Annexion der Krim, die Aggression in der Ostukraine und die Intervention in Syrien als Reaktion eines sich endlich „von seinen Knien“ erhebenden Russlands zu verstehen. Versailles wird darum zum bloßen historischen Klischee, um damit die geopolitischen Chimären des Kremls stärker herauszustellen und dabei aber gänzlich aus dem Blick zu verlieren, dass eine solche Sichtweise auf Versailles den deutschen Angriffs- und Vernichtungskrieg als gerechtfertigt erscheinen lässt. Zudem weist Putin mit diesem Brückenschlag zwischen Geschichte und Zeitgeschehen darauf hin, dass nur seine fortgesetzte Präsidentschaft verhindere, dass radikale Kräfte in Russland die Überhand gewännen, um sich damit gegenüber der Weltöffentlichkeit als alternativloser präsidialer Fels in der tobenden politischen Brandung zu inszenieren.

Abenteuerlich wird Putins Geschichtslektion, wenn er sich daran macht, die sowjetischen Annexionen durch den Hitler-Stalin-Pakt und die damit verbundenen Kriegshandlungen schon vor 1941 zu rechtfertigen. Während das militärische Argument, sich durch Truppenverlagerungen Richtung Westen in eine bessere strategische Position zu bringen, noch halbwegs nachvollziehbar wirkt, verblüffte Putin mit der im Baltikum für heftigen Protest sorgenden Aussage, die „Inkorporation Lettlands, Litauens und Estlands“ und „ihr Beitritt zur UdSSR“ seien „auf vertraglicher Basis unter Zustimmung der gewählten Obrigkeit“ und unter Beachtung der „Normen des Völker- und Staatsrechts der damaligen Zeit“ erfolgt. Tatsächlich handelte es sich 1940 um eine Annexion *par excellence* mit einem Ultimatum, einem gewaltsam eingesetzten Moskauer Marionettenregime und einer Scheinwahl.^106^ Die anschließende, von Terror und Massendeportationen begleitete Sowjetisierung verschweigt Putin genauso wie den sowjetischen Winterkrieg 1939/40 gegen Finnland, mit dem der Kreml massive Grenzverschiebungen zu seinen Gunsten erzwang und diese mit mehr als 200.000 toten und knapp 300.000 verwundeten Rotarmist_innen teuer erkaufte. Das Blutvergießen hatte bei den sowjetischen Streitkräften schon lange vor dem Angriff der Wehrmacht im Juni 1941 begonnen.^107^

Besonders verstört Putins Aussage, beim Einmarsch in Polen hätte die sowjetische Führung im September 1939 jederzeit die Möglichkeit gehabt, „die Westgrenze der UdSSR noch weiter nach Westen, bis nach Warschau zu verschieben“. Er greift damit eine neuerdings in der russischen Öffentlichkeit vermehrt zu vernehmende Drohgebärde auf, russische Streitkräfte könnten innerhalb weniger Tage erneut Kiew, Warschau und Berlin besetzen, wenn die dortigen Regierungen meinten, sich gegen Moskau stellen zu müssen. Putins sinisterem Fingerzeig kommt politische Brisanz zu, weil die polnische Staatsführung ihre guten Beziehungen zu Washington seit einiger Zeit nutzt, um die Verlegung von US-Truppen auf ihr Territorium zu erreichen. Darin sieht der Kreml einen Affront und lässt seitdem nichts unversucht, Polen als angeblichen Satellitenstaat der USA mit Säbelrasseln unter Druck zu setzen. Dieses tagesaktuelle Konfliktgeschehen hilft zu erklären, warum Putin seinen Abhandlungen zur Kriegsschuldfrage eine derart starke antipolnische Stoßrichtung gibt.

Statt die Heimsuchung der von der Roten Armee im September 1939 annektierten polnischen Territorien durch den stalinistischen Massenterror und den erbitterten polnischen Widerstand zu thematisieren,^108^ erklärt Putin, die vorrückenden sowjetischen Streitkräfte hätten die „verschiedenen Nationalitäten, darunter Juden“ vor der Vernichtung durch die „Nazis und ihre örtlichen Schergen“ gerettet.^109^ Unerwähnt bleibt dabei, dass die Sowjets bei den ethnischen und politischen „Flurbereinigungen“ der vormals polnischen Gebiete mit den Nazis gemeinsame Sache machten. Sie beteiligten sich aktiv an Umsiedlungen und Deportationen; zugleich nahmen sie Auslieferungen und Massenerschießungen (etwa in Katyn) vor, um die polnische Bevölkerung ihres Bürgertums sowie ihrer militärisch-politischen Elite zu berauben. Die sowjetischen Besatzer_innen agierten weniger als Retter_innen, sondern vielmehr als Vollstrecker_innen. Deren große Verbrechen sollen im Lichte der noch größeren Verbrechen Nazi-Deutschlands offensichtlich zum Zwecke der Relativierung in den Schatten gestellt werden.

In Putins Traktat fällt eine merkwürdige historische Leerstelle auf: dass nämlich die Eskalationsdynamik des Vernichtungskriegs und die gesamte sich daraus entwickelnde Zerstörung ihren Ursprung allein im rassistisch motivierten Eroberungswillen des nationalsozialistischen Regimes hatte. Putin geht es vor allem darum, Polen als Mitverursacher und nicht als Opfer des Zweiten Weltkriegs erscheinen zu lassen. Deshalb erwähnt er mit keiner Silbe, dass das Land in Hitlers menschenverachtenden Kriegs- und Großraumplänen von Beginn an zur brutalen Ausplünderung vorgesehen war und deshalb proportional mit dem Verlust eines Drittels seiner vormaligen Bevölkerung durch Tod und Vertreibung am meisten unter dem blutigen Weltkriegsgeschehen zu leiden hatte.^110^

Wie sehr Putin die Weltkriegsgeschichte missbraucht, um die aktuelle internationale Lage in seinem Sinne gestalten zu können, zeigt sich darin, dass er sein langes historisches Pamphlet mit einem seltsamen Appell ausklingen lässt. Es sei Zeit für ein neues Jalta, damit die fünf Vetomächte des UN-Sicherheitsrats – also die vier Siegermächte des Zweiten Weltkriegs plus China – gemeinsam die Weltordnung des 21. Jahrhunderts schaffen können. Diese befremdliche Nostalgie für eine Zeit, während der die Großmächte bei Gipfeltreffen Europa noch in Einflusszonen aufteilten und damit anderen Ländern eine nur „eingeschränkte Souveränität“ zubilligten, verdeutlicht ein rückwärtsgewandtes Verständnis von internationalen Beziehungen und zugleich eine äußerst selektive Deutung des Kalten Krieges. Völlig übergangen wird, dass die mit nuklearen *overkill*-Kapazitäten aufgeladene Blockrivalität, die permanente innergesellschaftliche Bedrohungskommunikation und die zahllosen blutigen Stellvertreterkriege den Kalten Krieg zum „radikalen Zeitalter“ werden ließen.^111^

Zudem ist kaum vorstellbar, dass sich die globalisierte, multilateral organisierte Welt des 21. Jahrhunderts in einem internationalen System organisieren ließe, dessen Gestaltung vor allem einigen wenigen Großmächten obliegt. Wirkmächtige supranationale Institutionen wie die EU, die neue Formen länderübergreifender Kooperation und Integration ermöglicht haben, sowie selbstbewusst agierende Staaten mit starken Eigeninteressen machen den Rückfall in die längst vergangene Welt der Großmächte unmöglich. Zudem lassen sich aktuelle Herausforderungen wie Klimawandel und Pandemien sowie die Organisation der weltumspannenden Zirkulation von Menschen, Waren und Ideen nur durch die Interaktion der gesamten Weltgemeinschaft sinnvoll lösen.

Der sonderbare, unzeitgemäße Traum von einem neuen Jalta dient Putin offensichtlich dazu, Russlands Anspruch als Weltordnungsmacht mit Nachdruck zu unterstreichen und die vormalige Moskauer Vormachtstellung in Osteuropa nicht in der Mottenkiste der Geschichte verschwinden zu lassen.^112^ Bei Putins Erwähnung von Jalta werden Beobachter_innen in den sich nach 1989 aus dem Moskauer Machtorbit gelösten Staaten vermutlich aufhorchen. Dort gilt das Gipfeltreffen als Verrat der Westmächte, weil diese als „Preis für den Frieden“ Stalin damals die Hegemonie über Osteuropa zugestanden hätten.^113^

## Putins Beschwörung eines deutsch-russischen Bilateralismus: 22. Juni 2021

Auch den nächsten bedeutsamen Gedenktag, als sich am 22. Juni 2021 der deutsche Überfall auf die Sowjetunion zum 80. Mal jährte, nutzte Putin erneut, um sich als selbstbewussten Geschichtserklärer mit großem Deutungsanspruch in Szene zu setzen. Der Redaktion der Wochenzeitung „Die ZEIT“ ließ er einen Beitrag zukommen, indem er – wieder ausgehend vom Zweiten Weltkrieg – seine Sicht auf Europas Geschichte und seine Vorstellung von einer gemeinsamen Zukunft ausbreitete. Erneut verwahrte sich Putin nachdrücklich gegen angebliche „jüngste Versuche, die Kapitel der Vergangenheit neu zu schreiben“. Die historische Wahrheit laute vielmehr, „dass der Sowjetsoldat seinen Fuß nicht auf deutschen Boden setzte, um sich an den Deutschen zu rächen, sondern um seine edle und große Befreiungsmission zu erfüllen“.^114^ Diese Sicht geht geflissentlich über die doppelte Gewalterfahrung hinweg, die viele Menschen im östlichen Europa sowohl durch den Nationalsozialismus als auch durch den Stalinismus zu ertragen hatten.

Weil sich der Beitrag explizit an ein deutsches Publikum wandte und offensichtlich bestehende russlandfreundliche Orientierungen bestärken wollte, vermied es Putin, von deutscher Schuld zu sprechen und deutsche Kriegsverbrechen zu benennen. Auch die Weltkriegsursachen thematisierte er dieses Mal nicht, um so einen Bogen um die mit dem Hitler-Stalin-Pakt verbundenen Kontroversen schlagen zu können. Durch diese Auslassungen blieb aber vieles im Vagen und Unbestimmten; der Zweite Weltkrieg erschien als diffuser Schicksalsschlag, der Europa ereilte, und nicht als historisches Ereignis mit konkreten Ursachen, Tätern und Folgewirkungen.

Mit seiner auf Versöhnung, Kriegsprävention und technologisch-wirtschaftliche Zusammenarbeit zielenden Rhetorik rief Putin erneut zu einem Neuanfang in den Beziehungen zwischen Russland und Europa auf. Dieses Mal richtete er seinen Appell nicht an die Siegermächte des Zweiten Weltkriegs, sondern allein an Deutschland. Das Dialogangebot war jedoch vergiftet, weil Putin die in konservativen sowie linken Milieus beliebte Vorstellung einer Berlin-Moskau-Achse als geopolitisches Gegengewicht zu den USA und China beschwor. In dieser Weltsicht erschienen die ostmitteleuropäischen Staaten wieder nur als bloße Verfügungsmasse der deutschen und russischen Politik. Deshalb konstruierte Putin ausgehend vom leidvollen Triumph des „sowjetischen Volks“ im Zweiten Weltkrieg ein imaginiertes „Wir“, dessen „untrennbaren kulturellen und geschichtlichen Bande zu Europa“ er rezitierte und damit den Anspruch erhob, nicht nur für Russland, sondern vielmehr für den gesamten vormaligen sowjetischen Raum zu sprechen. Dieser Bezug auf das historische „Wir“ von 1945 vermittelte die Vorstellung, dass Moskau im 21. Jahrhundert weiterhin das Recht auf eine eigene Einflusszone im östlichen Europa habe.

Mit seinem historisch hergeleiteten Mantra eines deutsch-russischen Bilateralismus bediente Putin geschickt die Gemütslage der deutschen Öffentlichkeit. Als Grundursache für den „desolaten Zustand“ der europäischen Sicherheitsordnung machte er allein das Vorrücken der NATO und der EU nach Osteuropa aus. Der damit verbundene Wandel der politischen Landkarte entsprach laut Putin nicht dem politischen Willen der ostmitteleuropäischen Staaten, sondern sei allein Folge der Machinationen und Interventionen westlicher Politik gewesen. In Rahmen seiner zeithistorischen Mythenbildung warf der Kremlchef den USA unverblümt vor, 2014 in der Ukraine einen „Staatsstreich“ organisiert zu haben, den die EU-Staaten „willenlos unterstützten und somit die Spaltung innerhalb der Ukraine und den Austritt der Krim aus dem ukrainischen Staat provozierten“.

Bei völliger Missachtung der Prinzipien von Demokratie und nationaler Selbstbestimmung zeigte Putin keinerlei Verständnis dafür, dass souveräne Staaten frei über die Wahl ihrer Bündnispartner entscheiden und die Majdan-Revolution 2014, die zu einem politischen Neuanfang in der Ukraine führte, Ausdruck des Volkswillens war. In seiner antiwestlichen Weltsicht ignorierte der russische Präsident völlig, dass sich die ostmitteleuropäischen Staaten von ihrer Zugehörigkeit zur NATO und EU mehr Sicherheit vor den imperialen Ambitionen im Kreml versprachen und es beim Sturz der Regierung von Wiktor Janukowytsch darum ging, die ukrainische Demokratie gegen ein zunehmend autoritär agierendes Regime zu verteidigen.^115^

## Schluss: Geschichtskonflikte statt Modernisierungserfolge

Als der deutsche Bundespräsident Richard von Weizsäcker am 8. Mai 1985 seine berühmte Rede zum Jahrestag des Weltkriegsendes hielt, erkannte er an, dass die europäische Erinnerungskultur von Geschichtskonflikten geprägt war, die sich aus den unterschiedlichen Erfahrungen der einzelnen Gesellschaften und Gruppen ergaben. Sein Anliegen war es, aus dem unversöhnlichen Gegeneinander ein produktives Miteinander zu machen. Differenzen sollten nicht zu einem einheitlichen, identitätspolitisch instrumentalisierbaren Geschichtsbild eingeschmolzen werden, so wie es einigen EU-Bürokrat_innen heute vorschwebt. Es gelte vielmehr, mit einem humanitären Begriff der rettenden Befreiung und dem eindringlichen Blick auf die Verwerfungen des Kriegsendes das vielseitige Ringen um die Geschichte aushaltbar zu machen, um über divergierende kollektive Gedächtnisse in einen verständnisvollen Dialog zu kommen.^116^

Als Geschichtspolitiker, der die Rolle des gesellschaftlichen Cliotherapeuten^123^ an sich gerissen hat, wandelt Putin weder auf Weizsäckers Spuren der Aussöhnung noch folgt er Daniil Granins Bemühungen, der platten Heroisierung durch die Aufarbeitung individueller Leiderfahrung und durch die kritische Thematisierung problematischer historischer Aspekte zu entkommen. Der russische Präsident übersieht geflissentlich, dass der Triumph im Zweiten Weltkrieg auf komplizierte, konfliktträchtige Weise gemeinsame europäische Geschichte und Erinnerung geworden ist. Stattdessen setzt er mit seinem polarisierenden Geschichtstraktat alles daran, im Gestus der Unerschütterlichkeit den Sieg am 9. Mai 1945 zum historischen Eigentum Russlands zu erklären und ihn in eine uneinnehmbare Festung der Erinnerungskultur und Identitätspolitik zu verwandeln. Das heroische Pathos von Größe, Mut und Aufopferung bestimmt darum seit einiger Zeit die Tonlage, in der die offiziellen Medien und die russische Regierung über den Krieg sprechen.

Äußerst bedenklich stimmt, dass der Kampf gegen vermeintliche „Geschichtsfälschung“ in Russland mittlerweile Verfassungsrang erhalten hat und dafür im Ermittlungskomitee der Generalstaatsanwalt im Sommer 2020 eine entsprechende Abteilung eingerichtet worden ist. Deren Aufgabe besteht allein darin, politisch missliebige Geschichtsdeutungen strafrechtlich zu verfolgen. Es bleibt abzuwarten, ob diese politische Droh- und Sanktionsmaßnahme zu einer weiteren Demontage der seriösen Forschung in Russland führt.^117^

Grund zur Sorge gibt zudem, dass die russischen Behörden im Vorfeld der Duma-Wahlen im September 2021 durch repressive Maßnahmen ihren Zugriff auf die Medienlandschaft und das Internet noch einmal massiv verstärkt haben. Der Druck des Kremls auf Internet-Provider, die Blockade von „GoogleDoc“ und die Sperrung kritischer Portale weisen genauso wie die heftigen Cyberattacken auf politisch engagierte Presseorgane daraufhin, dass der in Russland tobende „Informationskrieg“ gegen unliebsame Meinungen weiter verschärft wird. Dadurch dürfte es in Russland immer schwieriger werden, differenzierte und unvoreingenommene Geschichtsdiskussionen anzustoßen.^118^

Neben der innergesellschaftlichen Eintracht hat Putin die Spaltung Europas längst zum Lebenselixier seines autoritären Populismus und Nationalismus erhoben. Bei seiner Thematisierung der Kriegsschuldfrage im Juni 2020 folgt er darum dem Modus eines aggressiven *blame game*, um mit einem atemberaubenden Gespür für historische Bösartigkeiten andere Regierungen zu provozieren und auf geschichtspolitische Minenfelder zu locken. Die verstärkten verbalen Angriffe und Diffamierungen des Kremls lassen bereits abgekühlte Beziehungen noch kälter werden. Die teils infam verzerrende historische Propagandashow führt zur Verhärtung der Fronten und zur fortschreitenden Selbstisolierung Russlands. Im Juni 2021 schlug Putin mit seinen zeithistorischen Mythen gegenüber der deutschen Öffentlichkeit dann einen versöhnlichen Ton an, aber nur um mit seiner Idee von einem deutsch-russischen Bilateralismus und einem offensichtlichen Antiamerikanismus die moskaufreundlichen Stimmungen zu stärken.

Angesichts der 2020 vollzogenen Verfassungsänderungen, die dem russischen Staatsoberhaupt den weiteren Verbleib im Kreml garantieren, inszeniert sich Putin mit seinem neuen „history fetish“^119^ als tapfer kämpfender Retter der sowjetischen Weltkriegsgeschichte. Damit versucht er, in einer turbulenten Zeit davon abzulenken, dass die Corona-Pandemie die Gesellschaft tief erschüttert und die sich kaum mehr verbessernde sozioökonomische Situation Anlass zu wachsender Sorge gibt.^120^ Im Rahmen eines verbissenen „patriotism of despair“^121^ dient Geschichte keineswegs nur als politisches Dekor. Putins Appell, das heroische Erbe der Vorfahren zu wahren, erhöht seinen selbstherrlichen Kurs vielmehr zu einer historischen Mission. In einer Gesellschaft, deren wirtschaftliche Gegenwart und politische Zukunft ohne erfolgversprechendes Innovationsmodell nicht rosig erscheinen, stellt die Vergangenheit eine der wenigen nicht-mineralischen Ressourcen dar, über die Russland im Übermaß verfügt und die seine Polittechnokrat_innen in Zeiten wachsender Orientierungslosigkeit umso intensiver ausbeuten.^122^

Ein näherer Blick auf das osteuropäische Schlachtfeld der Erinnerungen zeigt, dass der geschichtspolitische Aktionismus mit seinen gezielten Salven nach innen und außen die wichtige Funktion hat, politischen Krisen und gesellschaftlichen Stillstand zu übertünchen. Neben Russland gilt auch für die vom Kreml geschichtspolitisch unter Beschuss genommenen Nachbarländer, dass Regierungen das historische Gedenken überschwänglich bemühen, wenn politische Erfolge ausbleiben und längst überfällige Reformprozesse nicht gelingen. Aggressive Geschichtspolitik dient dann als Surrogat für konstruktive Modernisierung.

Dabei ist zu beobachten, dass je mehr erinnerungskulturelle Konflikte die Gesellschaft mobilisieren und Politik legitimieren, es umso weniger Anreize und Notwendigkeiten dafür zu geben scheint, die eigentlichen Herausforderungen der Gegenwart und Zukunft anzugehen. Angesichts der aktuell in Osteuropa allerorten verabreichten Überdosis an Geschichte erweist sich eine erinnerungskulturelle Entziehungskur als unumgänglich, um sich durch Gespenster der Vergangenheit nicht davon ablenken zu lassen, die wirklichen gesellschaftlichen Probleme anzugehen.

## Auswahlbibliografie

- Bernsand, Niklas/Törnquist-Plewa, Barbara (Hrsg.): Cultural and Political Imaginaries in Putin’s Russia, 276 S., Brill, Leiden u. a. 2019.
- Becker, Anna: Mythos Stalin. Stalinismus und staatliche Geschichtspolitik im postsowjetischen Russland der Ära Putin, 158 S., be.bra, Berlin 2015.
- Gessen, Masha: Vergessen. Stalins Gulag in Putins Russland, 160 S., dtv, München 2019.
- Kolesnikov, Andrej: Erinnerung als Waffe. Die Geschichtspolitik des Putin-Regimes, in: Osteuropa 70 (2020), H. 6, S. 3–28.
- Ganzenmüller, Jörg/Utz, Raphael (Hrsg.): Sowjetische Verbrechen und russische Erinnerung. Orte – Akteure – Deutungen, 318 S., De Gruyter Oldenbourg, München 2014.
- Etkind, Alexander: Warped Mourning. Stories of the Undead in the Land of the Unburied, 328 S., Stanford UP, Stanford, CA 2013.
- Koposov, Nikolay: Memory Laws, Memory Wars. The Politics of the Past in Europe and Russia, 321 S., Cambridge UP, Cambridge 2017.
- Amacher, Korine/ Portnov, Andrii / Serhiienko, Viktoriia (Hrsg.): Official History in Eastern Europe, 362 S., Fibre, Osnabrück 2021.
- Gabowitsch, Mischa/Gdaniec, Cordula/Makhotina, Ekaterina (Hrsg.): Kriegsgedenken als Event. Der 9. Mai 2015 im postsozialistischen Europa, 345 S., Schöningh, Paderborn 2017.
- Yablokov, Ilya: Fortress Russia. Conspiracy Theories in the Post-Soviet World, 288 S., Polity, Cambridge 2018.
- Stewart, Susan: Geschichte als Instrument der Innen- und Außenpolitik am Beispiel Russlands. Wie die Gegenwart die Vergangenheit beeinflusst, in: SWP-Studie 22, November 2020, S. 1–40, URL: <https://www.swp-berlin.org/publications/products/studien/2020S22_geschichtspolitik_russland.pdf> [letzter Zugriff 17.11.2021].
- Kaminsky, Anna/Müller, Dietmar/Troebst, Stefan (Hrsg:): Der Hitler-Stalin-Pakt 1939 in den Erinnerungskulturen der Europäer, 566 S., Wallstein, Göttingen 2011.

## Footnotes

1 Gloger, Katja: Fremde Freunde. Deutsche und Russen – Die Geschichte einer schicksalhaften Beziehung, Piper, Berlin 2017, S. 239; Weller, Nina: Daniil Granin, in: Dekoder. Russland Entschlüsseln, URL: <https://www.dekoder.org/de/gnose/daniil-granin-blockadebuch> [letzter Zugriff am 05.10.2020].

2 Gräßler, Bernd: Bundestag gedenkt der Blockade Leningrads, in: Deutsche Welle, URL: <https://www.dw.com/de/bundestag-gedenkt-der-blockade-leningrads/a-17390306> [letzter Zugriff am 05.10.2020].

3 Schlögel, Karl: Marjampole oder Europas Wiederkehr aus dem Geist der Städte, Carl Hanser, München 2005, S. 171–182.

4 Eine aktuelle, ins Deutsche übersetzte Gesamtausgabe ist Adamowitsch, Ales/Granin, Daniil: Blockadebuch. Leningrad 1941–1944, aufbau, Berlin 2018 (auch Bonn 2019).

5 Weller, Nina: Vielstimmige Gegengeschichten. Kriegserfahrung und Kriegsdarstellung bei Ales’ Adamovič, Daniil Granin und Svetlana Aleksievič, in: Osteuropa 68 (2018), H. 1–2, S. 164–182. Zur Blockade vgl. ferner Sapper, Manfred/Weichsel, Volker (Hrsg.): Die Leningrader Blockade. Der Krieg, die Stadt und der Tod, in: Osteuropa 61 (2011), H. 8–9; Voronina, Tat’jana: Pomnit’ po-našemu. Socrealističeskij istorizm i blokada Leningrada, Novoe literaturnoe obozrenie, Moskva 2018; Weinmann, Daniel: Überlagertes Gedächtnis. Das Blockadebuch und geschichtspolitische Konstruktionen in der Sowjetunion (unveröffentlichte Bachelorarbeit), Tübingen 2020.

6 Granins unverhohlene Kritik an der Leningrader Elite griff Alexej Krasovskijs Film *Prazdnik* (Feiertag) auf, der 2019 von Abgeordneten der russischen Duma als „Blasphemie“ verurteilt wurde und als bitterböse Geschichtskomödie in den russischen Kinos floppte.

7 Wortprotokoll der Rede Wladimir Putins im Deutschen Bundestag am 25.09.2001, URL: <https://www.bundestag.de/parlament/geschichte/gastredner/putin/putin_wort-244966> [letzter Zugriff am 05.10.2020].

8 Dieses geschönte Geschichtsbild brachte der deutsche Politikwissenschaftler Heinrich Vogel schon 2001 auf die treffende Formel „Gagarin minus Gulag“. Vgl. Vogel, Heinrich: Charisma und Zweifel. Präsident Putin vor großen Herausforderungen, in: Internationale Politik (2001), H. 10, S. 7–16, hier S. 14.

9 Merridale, Catherine: Redesigning History in Contemporary Russia, in: Journal of Contemporary History 38 (2003), S. 13–28; de Keghel, Isabelle: Die Staatssymbolik des neuen Russland. Traditionen – Integrationsstrategien – Identitätsdiskurse, LIT, Münster 2009; Karl, Lars/Polianski, Igor J. (Hrsg.): Geschichtspolitik und Erinnerungskultur im neuen Russland, Vandenhoeck & Ruprecht, Göttingen 2009; Kalinin, Ilya: Nostalgic Modernization. The Soviet Past as „Historical Horizon“, in: Slavonica 17 (2011), H. 2, S. 156–166; Malinova, Olga: Constructing the „Usable Past“. The Evolution of the Official Historical Narrative in Post-Soviet Russia, in: Bernsand, Niklas/Törnquist-Plewa, Barbara (Hrsg.): Cultural and Political Imaginaries in Putin’s Russia, Brill Academic Pub, Leiden u.a. 2019, S. 85–104.

10 Poslanie Prezidenta RF V.V. Putina Federal’nomu Sobraniju RURF, 25 aprelja 2005 god, URL: <http://kremlin.ru/events/president/transcripts/22931> [letzter Zugriff am 05.10.2020].

11 Vinokurov, Evgenij/Göz, Roland: Unter Partnern. Die Eurasische Wirtschaftsunion, in: Osteuropa 66 (2016), H. 5, S. 129–140; Kukulin, Il’ja: „Eurasien“ statt „Russische Welt“. Zum Wandel eines schillernden Begriffs, in: Osteuropa 67 (2017), H. 11–12, S. 173–186; Vinokurov, Evgeny: Introduction to the Eurasian Economic Union, palgrave macmillan, London 2018**.**

12 The Putin Interview: Oliver Stone Interviews Vladimir Putin, New York 2017.

13 Becker, Anna: Mythos Stalin. Stalinismus und staatliche Geschichtspolitik im postsowjetischen Russland der Ära Putin, be.bra, Berlin 2016, S. 117.

14 Ebd., S. 103 f.

15 Vgl. z. B. die Ergebnisse der vom *Levada Zentrum* durchgeführten repräsentativen Umfragen Analitičeskij centr Jurija Levady: Obščestvennoe mnenie 2019. Ežegodnik, Moskva 2019, S. 174, URL: <https://www.levada.ru/2017/02/15/lyubov-rossiyan-k-stalinu-dostigla-maksimuma> [letzter Zugriff am 05.10.2020]. Ferner Kaminskij, Konstantin: Stalin-Kult 2.0. Stalin-Kult in russischen Medien des 21. Jahrhunderts, in: Forum für osteuropäische Ideen- und Zeitgeschichte 16 (2012), H. 1, S. 165–187; Kurilla, Ivan: The „Return of Stalin“. Understanding the Surge of Historical Politics in Russia, in: PONARS Eurasia Policy Memo No. 429, May 2016, S. 1–6; Khlevnyuk, Daria: Narrowcasting Collective Memory Online. ‚Liking‘ Stalin in Russian Social Media, in: Media, Culture & Society 41 (2019) H. 3, S. 1–15; Perrier, Elena: Memory Watchdogs. Online and Offline Mobilizations around Controversial Historical Issues in Russia, in: Bernsand: Imaginaries (wie Anm. 9), S. 141–174; Ėpple, Nikolaj: Neudobnoe prošloe. Pamjat’ o gosudarstvennych prestuplenijach v Rossii i drugich stranach, Moskva 2020, S. 18 f; Frieß, Nina: Der Solowezki-Stein, in: Dekoder. Russland Entschlüsseln, 05.10.2021, URL: <https://kremlin.dekoder.org/solowezki-stein> [letzter Zugriff am 22.10.2021].

16 Giesen, Anke: „Wie kann denn der Sieger ein Verbrecher sein?“ Eine diskursanalytische Untersuchung der russlandweiten Debatte über Konzept und Verstaatlichungsprozess der Lagergedenkstätte „Perm’-36“ im Ural, Ibidem, Stuttgart 2019; Sniegon, Tomas: Dying in the Soviet Gulag for the Future Glory of Mother Russia. Making „Patriotic“ Sense of the Gulag in Present-Day Russia, in: Bernsand: Imaginaries (wie Anm. 9), S. 105–140.

17 Rebitschek, Immo: Neuvermessung und Neugestaltung eines Erinnerungsortes. Die Gedenkstatte Perm’-36, in: Ganzenmüller, Jörg/Utz, Raphael (Hrsg*.*): Sowjetische Verbrechen und russische Erinnerung. Orte – Akteure – Deutungen, De Gruyter, München 2014, S. 91–108; Klymenko, Lina: „Perm-36“. Die umkämpfte russische Erinnerung an die politischen Repressionen in der Sowjetunion, in: Russland-Analysen Nr. 368 (15.03.2019), S. 5–8. Zu den Ausstellungsstrategien in GULag-Museen vgl. allgemein Sniegon: Gulag (wie Anm. 16); Khlevnyuk, Daria: Victims’ Categorizations. Making Sense of Purges in Karelia and on Solovki, in: Laboratorium. Russian Review of Social Research 10 (2018), H. 2, S. 122–33.

18 Gessen, Masha: Vergessen. Stalins Gulag in Putins Russland, dtv, München 2019, S. 105 f.

19 Meduševskij, Andrej: Was war der Stalinismus. Die Stalinismus-Edition des Verlags ROSSPEN, in: Osteuropa 62 (2012) H. 4, S. 53–60.

20 Chlevnjuk, Oleg: Stalin. Žizn’ odnogo voždja, Exlibrus, Moskva 2015 (deutsche Ausgabe: Stalin. Eine Biographie, Siedler, München 2015).

21 Zu dieser Umfrage vgl. Kolesnikov, Andrej: Erinnerung als Waffe. Die Geschichtspolitik des Putin-Regimes, in: Osteuropa 70 (2020), H. 6, S. 3–28, hier S. 26 f.

22 Beispiele dafür sind z. B. Irina Ščerbakova, Leonid Parfenov, Vladimir Pozner, Boris Akunin, Ljudmila Ulickaja, Alexej Pivorov, der Sender *Dožd’*, das Radioprogramm *Echo Moskvy*, die Zeitung *Novaja Gazeta*, das *Namedni*-Projekt, der Redacija-Kanal auf *YouTube* und Internet-Portale wie URL:<https://www.colta.ru/>, URL: <https://diletant.media/kartoteka/>, URL: <https://arzamas.academy/courses> und URL: <https://meduza.io/specials/bezussr> [letzter Zugriff am 11.11.2021]. Sogar im kulturorientierten, werbefreien Staatssender *Rossija K* (bis 2009 *Kul’tura*) laufen immer wieder professionelle historische Dokumentationen. Vgl. Perrier, Elena: Memory Watchdogs, in: Bernsand: Imaginaries (wie Anm. 9), S. 141–174; Kuntsman, Adi: Online Memories, Digital Conflicts and the Cybertouch of War, in: Digital Icons: Studies in Russian, Eurasian and Central European New Media 4 (2010), S. 1–12; Rutten, Ellen/Fedor, Julie/Zvereva, Vera (Hrsg.): Memory, Conflict and New Media. Web Wars in Post-Socialist States, Taylor & Francis, London 2013; Werkmeister, Christian/Müller-Butz, Martin: Die Geschichte des GULag im RuNet. Möglichkeiten und Grenzen virtueller Erinnerungskulturen, in: Ganzenmüller: Verbrechen (wie Anm. 17), S. 217–244; Gorham, Michael u. a. (Hrsg.): Digital Russia. The Language, Culture and Politics of New Media Communication, Routledge, New York 2014; Gorbachev, Oleg: The *Namedni* Project and the Evolution of Nostalgia in Post-Soviet Russia, in: Canadian Slavonic Papers 57 (2015), H. 3–4, S. 180–94; Chut, Ljudmila R.: Istoričeskaja blogsfera Runet, URL: <https://cyberleninka.ru/article/n/istoricheskaya-blogosfera-runeta/viewer> [letzter Zugriff am 05.10.2020].

23 Zum Lagerkomplex an der Kolyma vgl. Sprau, Mirjam: Kolyma nach dem GULAG. Entstalinisierung im Magadaner Gebiet 1953–1960, De Gruyter Oldenbourg, Berlin 2018.

24 Der Dokumentarfilm ist online abrufbar, URL: <https://www.youtube.com/watch?v=oo1WouI38rQ> [letzter Zugriff am 24.03.2021].

25 Vgl. zu den medialen Debatten Ėpple: Neudobnoe prošloe, (wie Anm. 15), S. 17 f.; Schor-Tschudnowskaja, Anna: A Pop-Cultural Reappraisal of State Terror. The YouTube Film ‚Kolyma—Birthplace of Our Fear‘ and Its Viewers, in: Cultures of History Forum, 29.04.2020, URL: <https://www.cultures-of-history.uni-jena.de/debates/russia/a-pop-cultural-reappraisal-of-state-terror-the-youtube-film-kolyma-birthplace-of-our-fear-and-its-viewers> [letzter Zugriff am 24.03.2021]; Debattenschau Nr. 76: Kolyma – Heimat unserer Angst, in: Dekoder. Russland Entschlüsseln, 22.05.2019, URL: <https://www.dekoder.org/de/article/debattenschau-kolyma-juri-dud-doku> [letzter Zugriff am 24.03.2021]. Die Zitate stammen allesamt aus der Debattenschau von Dekoder.

26 Vgl. dazu die Homepage des Museums, URL: <https://gmig.ru/> [letzter Zugriff am 14.10.2020]; Blick ins neue Gulag-Museum in Moskau, https://www.diepresse.com/5015071/blick-ins-neue-gulag-museum-in-moskau#slide-2 [letzter Zugriff am 24.03.2021]; Sniegon: Gulag, S. 124–130; Holm, Kerstin: Im Moskauer Gulag-Museum. Für die Verhaftungen gab es Planziffern, in: Frankfurter Allgemeine Zeitung, 05.05.2016, URL: <https://www.faz.net/aktuell/feuilleton/im-moskauer-gulag-museum-fuer-die-verhaftungen-gab-es-planziffern-14207025.html> [letzter Zugriff am 24.03.2021].

27 Ėpple: Neudobnoe prošloe, (wie Anm. 15), S. 52 f.; Voswinkel, Johannes: Operation „Letzte Adresse“, in: Die ZEIT (2014), Nr. 53, URL: <https://www.zeit.de/2014/53/moskau-erinnerung-opfer-stolpersteine> [letzter Zugriff am 14.10.2020]; Gabowitsch, Mischa: Foils and Mirrors. The Soviet Intelligentsia and German Atonement, in: ders. (Hrsg.): Replicating Atonement. Foreign Models in the Commemoration of Atrocities, Palgrave Macmillan, Basingstoke 2017, S. 267–304; Veselov, Fedor D.: Poslednij Adres’. Negosudarstvennyj memorial’nyj proekt i politika pamjati v Rossii, in: Miller, Alexej I./Efremenko, Dmitrij V. (Hrsg.): Politika pamjati v sovremennoj Rossii i stranach vostočnoj Evropy, Izdatelstvo Evropejskogo, Sankt-Peterburg 2020, S. 202–229. Gegen die Bürgeraktion regt sich mitunter auch Widerstand. Vgl. zu einem viel beachteten Beispiel in St. Petersburg bes. Dom vygljadit kak kladbiše, URL: https://meduza.io/feature/2020/10/19/dom-vyglyadit-kak-kladbische> [letzter Zugriff am 07.11.2020]. An der Helmut-Schmidt-Universität in Hamburg forscht Melanie Hussinger im Rahmen ihres Promotionsprojekts zu dieser Bürgeraktion.

28 Vgl. dazu die Homepage des Projekts, URL: <https://www.memo.ru/en-us/projects/topos> [letzter Zugriff am 14.10.2020].

29 Holm, Kerstin: Russischer Kampf ums Gedenken. Schießstand auf dem Massengrab, in: FAZ.NET, 10.01.2021, URL: <https://www.faz.net/1.7136660> [letzter Zugriff am 13.01.2020].

30 Luxmoore, Matthew: Moscow’s Stalin-Themed Kebab Shop Purged Within 24 Hours Of Launch, in: Radio Free Europe/Radio Liberty, 11.01.2021, URL: <https://www.rferl.org/a/russia-moscow-kebab-stalin-public-outcry/31042334.html> [letzter Zugriff am 17.11.2021]. Um die Geschichtsaktivist_innen von *Memorial* wegen ihres lautstarken Protests am *Stalin Döner* zu maßregeln, versammelten sich einige Tage später aufgebrachte Gemüter der patriotischen Vereinigung *Za pravdu* (Für die Wahrheit) vor dem Gebäude, in dem *Memorial *seine Büros hat. Sie drängten auf Einlass und echauffierten sich lautstark über die ihrer Meinung ungebührliche Kritik der „ausländischen Agenten“ an Stalin. Diese aggressionsgeladene Szene schien bangen Beobachter_innen einen ersten Vorgeschmack auf drohende Pogrome gegen diejenigen zu geben, die sich gegen die Apologie des Stalinismus stemmen. Vgl. Preljudija k pogromam, in: Novaja Gazeta, 11.01.2021, URL: <https://novayagazeta.ru/articles/2021/01/11/88657-prelyudiya-k-pogromam> [letzter Zugriff am 21.03.2021].

31 Ėpple: Neudobnoe prošloe, (wie Anm. 15), S. 50 f.; Putin fordert Schlussstrich unter Stalin-Ära, in: Deutsche Welle, 30.10.2017, URL: <https://p.dw.com/p/2ml7i> [letzter Zugriff am 09.01.2020]; Grieß, Thielko: Erinnerung mit Widersprüchen, in: Deutschlandfunk, 30.10.2017, URL: <https://www.deutschlandfunk.de/verfolgte-der-sowjet-diktatur-erinnerung-mit-widerspruechen.1773.de.html?dram:article_id=399415> [letzter Zugriff am 09.01.2020].

32 Gefter, Michail: „Stalin ist erst gestern gestorben …“, in: Afanassjew, Juri (Hrsg.): Es gibt keine Alternative zu Perestroika. Glasnost, Demokratie, Sozialismus, Greno, Nördlingen 1988, S. 379–412.

33 Etkind, Alexander: Warped Mourning. Stories of the Undead in the Land of the Unburied, Stanford UP, Stanford, CA 2013.

34 Becker: Mythos, (wie Anm. 13), S. 86**–**101; Koposov, Nikolay: Memory Laws, Memory Wars. The Politics of the Past in Europe and Russia, Cambridge UP, Cambridge 2017, S. 238**–**299.

35 Zum international vielbeachteten Eklat entwickelte sich vor allem die Justizfarce um den karelischen Historiker Jurij Dmitriev. Vgl. Stewart, Susan: Geschichte als Instrument der Innen- und Außenpolitik am Beispiel Russlands. Wie die Gegenwart die Vergangenheit beeinflusst, in: SWP-Studie 22, November 2020, S. 1–40, hier S. 27 f.; Lebedev, Sergej: Das Zeugnis der Toten. Jurij Dmitriev, Wanderer im Archipel, in: Osteuropa 67 (2017), H. 9–10, S. 3–15; Burtin, Schura: Die Geister der Vergangenheit, in: Dekoder. Russland Entschlüsseln, 30.05.2017, URL: <https://www.dekoder.org/de/article/dimitrijew-terror-solowezki-gulag> [letzter Zugriff am 00.10.2020]; Schmidt, Friedrich: Der Stachel im russischen System, in: Frankfurter Allgemeine Zeitung, 30.09.2020, URL: <https://www.faz.net/aktuell/politik/ausland/stalinismus-forscher-warum-jurij-dmitrijew-verurteilt-wurde-16979242.html?printPagedArticle=true#pageIndex_2> [letzter Zugriff am 20.10.2020].

36 Aktuell steht „Memorial“ vor Gericht. Der Organisation droht ein Verbot und die baldige Schließung, um es als gesellschaftliches Gegengedächtnis endgültig auszuschalten. Vgl. Oleg Kaschin: Die Liquidierung des Gewissens, in: Dekoder. Russland Entschlüsseln, 12.11.2021, URL: <https://www.dekoder.org/de/article/memorial-liqudierung-ngo-repressionen> [letzter Zugriff am 24.11.2021]; Lezina, Evgenija: Memorial und seine Geschichte. Russlands historisches Gedächtnis, in: Osteuropa 64 (2014), H. 11–12, S. 165–176; Ochotin, Grigorij: Agentenjagd. Die Kampagne gegen NGOs in Russland, in: Osteuropa 65 (2015), H. 1–2, S. 83–94; Ščerbakova, Irina: Memorial unter Druck. Techniken des repressiven Staates in Russland, in: Osteuropa 70 (2020), H. 3–4, S. 215–228; Golova, Tatiana: Weitere Verschärfung der russischen Gesetze über ausländische Agent_innen, in: ZOiS Spotlight 7/2021, 24.02.2021, URL: <https://www.zois-berlin.de/publikationen/weitere-verschaerfung-der-russischen-gesetze-ueber-auslaendische-agentinnen> [letzter Zugriff am 20.03.2021].

37 Becker: Mythos, (wie Anm. 13), S. 73–85.

38 Balmforth, Tom: Critics Of Russia’s New Education Minister See Threat To Secular Values, in: Radio Free Europe/Radio Liberty, 31.08.2016, URL: <https://www.rferl.org/a/russia-education-minister-vasilyeva-stalin-orthodox-values/27958314.html> [letzter Zugriff am 16.10.2020].

39 Becker: Mythos, (wie Anm. 13), S. 46–72; Brandenberger, David: A New Short Course. A. V. Filippov and the Russian State’s Search for a ‚Usable Past‘, in: Kritika 10 (2009), H. 4, S. 825–833; ders.: Promotion of a Usable Past. Official Efforts to Rewrite Russo-Soviet History, 2000–2014, in: Tismaneanu, Vladimir/Iacob, Bogdan C. (Hrsg.): Remembrance, History, and Justice. Coming to Terms with Traumatic Pasts in Democratic Societies, Central European UP, Budapest u.a. 2015, S. 191–212; Nelson, Todd H.: History as Ideology. The Portrayal of Stalinism and the Great Patriotic War in contemporary Russian High School Textbooks, in: Post-Soviet Affairs 31 (2015), H. 1, S. 37–65; Filina, Olga: Vom Versuch der „einheitlichen“ Geschichte (2019), in: Dekoder. Russland Entschlüsseln, URL: <https://www.dekoder.org/de/article/schulbuch-vereinheitlichung-geschichte-stalin> [letzter Zugriff am 05.10.2020]; Amacher, Korine: History Textbooks in Russia (1992–2019). Between Multisided and Imperial Perspectives, in: dies./Portnov, Andrii/Serhiienko, Viktoriia (Hrsg.): Official History in Eastern Europe, Fibre, Osnabrück 2020, S. 105–134.

40 Ėpple: Neudobnoe prošloe, (wie Anm. 15), S. 49; Rietzschel, Antonie: Wie Russland sich die Geschichte zurechtbiegt, in: Süddeutsche Zeitung, 22.11.2015, URL: <https://www.sueddeutsche.de/politik/moskau-wie-russland-sich-die-geschichte-zurechtbiegt-1.2742497> [letzter Zugriff am 14.10.2020]; Holm, Kerstin: Propaganda für die Interessen des Staates, in: Frankfurter Allgemeine Zeitung, 13.11.2017, URL: <https://www.faz.net/aktuell/feuilleton/russland-inszeniert-sich-seine-geschichte-schoen-15289169.html> [letzter Zugriff am 14.10.2020]; Nogovicyn, Grigorij: Leviafan na asfal’toukladčike. Čto predstavljaet soboj park „Rossija – moja istorija“, in: Zvezda, 11.05.2017, URL: <http://zvzda.ru/columns/1a611a48b24b> [letzter Zugriff am 14.10.2020]; Petrone, Karen: The 21st-Century Memory of the Great Patriotic War in the „Russia—My History“ Museum, in: Hoffmann, David L. (Hrsg.): The Memory of the Second World War in Soviet and Post-Soviet Russia (im Erscheinen), Online-Version, URL: <https://jordanrussiacenter.org/news/vladimir-putin-and-the-great-patriotic-war-in-the-russia-my-history-museum/#.YXJUBRx> [letzter Zugriff am 23.10.2020].

41 Kolesnikov: Erinnerung (wie Anm. 21), S. 3.

42 Jablokov, Il’ja: Feinde, Verräter, Fünfte Kolonnen. Verschwörungstheorien in Russland, in: Osteuropa 65 (2015), H. 5, S. 99**–**114; Yablokov, Ilya: Fortress Russia. Conspiracy Theories in the Post-Soviet World, Polity, Cambridge 2018.

43 Makhotina, Ekaterina: Der Große Vaterländische Krieg in der Erinnerungskultur, in: Dekoder. Russland Entschlüsseln, URL: <https://www.dekoder.org/de/gnose/erinnerungskultur-grosser-vaterlaendischer-krieg-sowjetunion> [letzter Zugriff am 10.10.2020]; Markova, Maria: The Political Use of Soviet Nostalgia to Develop a Russian National Identity, in: E‑International Relations, 14. Juli 2020, URL: < https://www.e-ir.info/pdf/86159> [letzter Zugriff am 10.10.2020]; Norris, Stephen M.: The War Film and Memory Politics in Putin’s Russia, in: Hoffmann, David L. (Hrsg.): The Memory of the Second World War (erscheint 2022 bei Routledge).

44 Bljum, Arlen: Blockierte Wahrheit. Blockadeliteratur und Zensur, in: Osteuropa 61 (2011), H. 8–9, S. 297–307, hier S. 307.

45 Deriglazova, Katya: Patriotisches Vergnügen, in: Dekoder. Russland Entschlüsseln, URL: <https://www.dekoder.org/de/article/park-patriot-hauptkirche-streitkraefte> [letzter Zugriff am 12.10.2020]; Medvedev, Sergej: Geschichte als Rummelplatz, in: Dekoder. Russland Entschlüsseln, URL: <https://www.dekoder.org/de/article/geschichte-krieg-terror-erinnerung> [letzter Zugriff am 12.10.2020]; Walker, Shaun: Vladimir Putin Opens Russian „Military Disneyland“ Patriot Park, in: The Guardian, 16. Juni 2015, URL: <https://www.theguardian.com/world/2015/jun/16/vladimir-putin-opens-russian-military-disneyland-patriot-park> [letzter Zugriff am 12.10.2020].

46 McGlynn, Jade: Militärgeschichte für die russische Jugend, in: ZOiS Spotlight 38/2020 (21. Oktober 2020), URL: <https://www.zois-berlin.de/publikationen/zois-spotlight/militaergeschichte-fuer-die-russische-jugend/> [letzter Zugriff am 22.10.2020].

47 Krawatzek, Félix: Geschichtsloyal? Wie die Jugend in Russland die Geschichte bewertet, in: ZOiS Spotlight 39/2018 (14.11.2018), URL: <https://www.zois-berlin.de/publikationen/zois-spotlight-2018/geschichtsloyal-wie-die-jugend-in-russland-die-geschichte-bewertet/> [letzter Zugriff am 16.10.2020]; ders./Frieß, Nina: World War II for Young Russians. The Production and Reception of History in: ZOiS Report, Nr. 1/2020 (April), URL: <https://www.zois-berlin.de/publikationen/zois-report/zois-report-12020/> [letzter Zugriff am 16.10.2020].

48 Auch in Kirchenkreisen greift seit einiger Zeit die Verehrung Stalins um sich. Beispielsweise versuchte 2008 eine russisch-orthodoxe Gemeinde in St. Petersburg, Stalin heilig zu sprechen. Auch wenn dieser Verstoß damals scheiterte, so gibt es seitdem immer wieder Ikonen mit Stalin. Vgl. Margolina, Sonja: Der heilige Josef Wissarionowitsch, in: Die WELT, 17.12.2008, URL: <https://www.welt.de/welt_print/article2890373/Der-heilige-Josef-Wissarionowitsch.html> [letzter Zugriff am 07.11.2020].

49 Kolesnikov: Erinnerung (wie Anm. 21), S. 9; Esch, Christian: Putin, Stalin und die Gottesmutter, in: Der SPIEGEL, 9. Mai 2020, URL: <https://www.spiegel.de/politik/ausland/zweiter-weltkrieg-75-jahre-kriegsende-wladimir-putin-josef-stalin-und-die-gottesmutter-a-3a432b9a-8f04-4f72-b7e9-7b41ee9a98ee> [letzter Zugriff am 05.10.2020].

50 Grossman, Wassili: Leben und Schicksal, Ullstein, München 1984, S. 686.

51 Becker: Mythos, (wie Anm. 13), S. 79 f.

52 Medvedev, Sergey: „Regen“ und Zensurhagel. Der unabhängige Fernsehsender „Doschd“ („Regen“) steht wegen einer Umfrage zur Leningrader Blockade vor dem Aus, in: Russland-Analysen Nr. 271 (14.02.2014), S. 15–16; Stewart: Geschichte (wie Anm. 35), S. 31 f. Sechs Jahre später thematisierte das russische Geschichtsmagazin *Diletant* im April 2020 erneut diese Frage, um auf einer sachlichen Ebene darüber zu diskutieren. Dass dieses Mal größere Empörung ausblieb, ist neben der Art der Diskussion vermutlich auch auf die begrenzte Reichweite des digitalen Mediums zurückzuführen. Vgl. Weinmann: Gedächtnis (wie Anm. 5), S. 50.

53 Stewart: Geschichte (wie Anm. 35), S. 27 f.; Klimeniouk, Nikolai: Kriegsdenkmäler in Russland. Die unfassbare Lüge dieser Helden, URL: <https://www.faz.net/aktuell/feuilleton/debatten/luege-ueber-kriegsdenkmaeler-in-russland-empoert-russen-13720292.html> [letzter Zugriff am 14.10.2020].

54 Scherbakowa, Irina: Zerrissene Erinnerung. Der Umgang mit Stalinismus und Zweitem Weltkrieg im heutigen Russland, Wallstein, Göttingen 2010; dies.: Die Hände meines Vaters. Eine russische Familiengeschichte, Droemer, München 2017.

55 Poljan, Pavel M.: Istoriomor, ili trepanacija pamjati. Bitvy za pravdu o GULAGe, deportacijach, vojne i Cholokoste, AST, Moskva 2016.

56 Klein, Eduard/Himmelsbach, Anton: St. Georgs-Band, in: Dekoder. Russland entschlüsseln, URL: <https://www.dekoder.org/de/gnose/st%C2%A0-georgs-band> [letzter Zugriff am 14.10.2020].

57 Demmel, Vera: Das Georgsband. Ruhmesorden, Erinnerungszeichen, Pro-Kreml-Symbol, in: Osteuropa 66 (2016), H. 3, S. 19–31; Gdaniec, Cordula: „Danke Opa! Ich bin in Berlin!“. Gedenken an den 9. Mai 1945. Postsowjetische Praktiken und Ort, in: Klei, Alexandra/Stoll, Katrin/Wienert, Annika (Hrsg.): 8. Mai 1945. Internationale und interdisziplinäre Perspektiven, Neofelis, Berlin 2016, S. 189–209, hier S. 200–205; Hampl, Stefan/Mihalits, Dominik: Russian Revival of the St. George Myth and Its Imagery, in: Wagoner, Brady u. a. (Hrsg.): The Psychology of Imagination. History, Theory and New Research Horizons, Information Age Publishing, Charlotte 2017, S. 295–318.

58 Ähnliche Aktionen hatte es schon 1965 in Novosibirsk und 2007 in Tjumen gegeben.

59 Vgl. die Homepage, URL: <https://www.moypolk.ru/> [letzter Zugriff am 14.10.2020]. Ferner Stewart: Geschichte (wie Anm. 35), S. 24 f.; Bilalutdinov, Azat: Die Gedenkinitiative „Unsterbliches Regiment“ zwischen Gesellschaft und Politik, in: Gabowitsch, Mischa/Gdaniec, Cordula/Makhotina, Ekaterina (Hrsg.): Kriegsgedenken als Event. Der 9. Mai 2015 im postsozialistischen Europa, Schöningh, Paderborn 2017, S. 126–140.

60 Von einer „idiotischen Initiative“ spricht der russische Historiker Alexej Miller: Wir zerstören, was wir erreicht haben, in: Dekoder. Russland Entschlüsseln, 23.04.2018, URL: <https://www.dekoder.org/de/article/wir-zerstoeren-was-wir-erreicht-haben> [letzter Zugriff am 15.10.2020]. Eine andere Sichtweise vertritt Alexander Etkind im Interview mit Alexander Gorbatschow, in: Dekoder. Russland Entschlüsseln, 09.05.2018, URL: <https://www.dekoder.org/de/article/krieg-erinnerung-etkind-aufarbeitung> [letzter Zugriff am 15.10.2020].

61 Die deutsche Übersetzung dieser Rede findet sich in Osteuropa 64 (2014), H. 5**–**6, S. 87**–**99.

62 Siegert, Jens: Notizen aus Moskau. Regime ohne Zukunft. Wie Russland zum Sieger im Ersten Weltkrieg wurde, in: Russland-Analysen, Nr. 286 (21.11.2014), S. 28–29; Dornblüth, Gesine: Vergessener Krieg. In Moskau wurde ein Denkmal für den Ersten Weltkrieg eingeweiht, URL: <http://www.deutschlandfunkkultur.de/vergessener-krieg-spaete-aufarbeitung.1013.de.html?dram:article_id=293421> [letzter Zugriff am 15.10.2020].

63 Makhotina, Ekaterina: Keine Experimente. Russlands Geschichtspolitik und die Revolution, in: Osteuropa 67 (2017), H. 6–8, S. 211–230; Kalinin, Il’ja: Antirevolutionäre Revolutionserinnerungspolitik. Russlands Regime und der Geist der Revolution, in: Osteuropa 67 (2017), H. 6–8, S. 7–17; Kurilla, Ivan: History and Memory in Russia during the 100-Year Anniversary of the Great Revolution, in: PONARS Eurasia Policy Memo Nr. 503, Januar 2018, S. 1–6.

64 de Keghel, Isabelle: Verordneter Abschied von der revolutionären Tradition. Der „Tag der nationalen Einheit“ in der Russländischen Föderation, in: Karl: Geschichtspolitik (wie Anm. 9), S. 119–140; Plamper, Jan: Erinnerung und Verdrängung der Revolution in Russland. Zwischen Märtyrologie, Konspirologie und starkem Staat, in: Behrends, Jan C. u. a. (Hrsg.): 100 Jahre Roter Oktober. Zur Weltgeschichte der Russischen Revolution, Links, Berlin 2017, S. 279–294.

65 Kolesnikov: Erinnerung (wie Anm. 21), S. 26.

66 Schmid, Ulrich: Technologien der Seele. Vom Verfertigen der Wahrheit in der russischen Gegenwartskultur, Suhrkamp, Berlin 2015.

67 Kalmbach, Cordula: Das Massaker erinnern. Katyń als lieu de mémoire der polnischen Erinnerungskultur, Peter Lang, Frankfurt a.M. 2015; Urban, Thomas: Katyn 1940. Geschichte eines Verbrechens, Beck München 2015; Weber, Claudia: Krieg der Täter. Die Massenerschießungen von Katyń, HIS, Hamburg 2015.

68 Kolb, Matthias: Estnischer Denkmalstreit. Blumen und Bananen, in: Süddeutsche Zeitung, 17.05.2015, URL: <https://www.sueddeutsche.de/kultur/estnischer-denkmalstreit-blumen-und-bananen-1.434220> [letzter Zugriff am 14.10.2020].

69 Spolitis, Veiko: Der estnische Denkmalstreit und die Beziehungen zwischen Russland und den baltischen Staaten, in: Russland-Analysen, Nr. 134 (11.05.2007), S. 2–4; Brüggemann, Karsten: Denkmäler des Grolls. Estland und die Kriege des 20. Jahrhunderts, in: Osteuropa 58 (2008), H. 6, S. 29–146; ders.: Geteilte Geschichte als transnationales Schlachtfeld, in: Hofmann, Birgit u. a. (Hrsg.): Diktaturüberwindung in Europa. Neue nationale und transnationale Perspektiven, UV Winter, Heidelberg 2010, S. 211–225; Lehti, Marko u. a.: Never-Ending Second World War. Public Performances of National Dignity and the Drama of the Bronze Soldier, in: Journal of Baltic Studies 39 (2008), H. 4, S. 393–418; Perchoc, Philippe: European Memory beyond the State. Baltic, Russian and European Memory Interactions (1991–2009), in: Memory Studies 12 (2019), H. 6, S. 677–698.

70 Yablokov: Fortress (wie Anm. 42).

71 Voswinkel, Johannes: Zynismus mit journalistischem Antlitz. Russlands Medien, die Macht und die Ukraine, in: Osteuropa 64 (2014), H. 5–6, S. 175–192; McGlynn, Jade: Performing the Great Patriotic War through the Ukraine Crisis, in: Memory Studies, 2018, 27.09.2018, URL: <10.1177/1750698018800740> [letzter Zugriff am 14.10.2020]; Makhortykh, Mykola: Remediating the Past. YouTube and Second World War Memory in Ukraine and Russia, in: Memory Studies 13 (2020), H.2, S. 146–161.

72 Vladimir Putin: Ob istoričeskom edinstve russkich i ukraincev, 12.07.2021, URL: <http://kremlin.ru/events/president/news/66181> [letzter Zugriff am 25.09.2021].

73 Eggert, Konstantin: Putins Kampf gegen das „Anti-Russland“, 20.07.2021, in: Dekoder. Russland Entschlüsseln, URL: <https://www.dekoder.org/de/article/putin-ukraine-aufsatz> [letzter Zugriff am 25.09.2021]; Goncharenko, Roman: Putin formuliert Ukraine-Doktrin und droht, in: Deutsche Welle, 15.07.2021, URL: <https://p.dw.com/p/3wXS> [letzter Zugriff am 25.09.2021]; Klimeniouk, Nikolai: Putin über die Ukraine. Wie im Hospiz, in: Frankfurter Allgemeine Zeitung, 17.07.2021, URL: <https://www.faz.net/aktuell/feuilleton/putin-ueber-die-ukraine-zurschaustellung-der-eigenen-unbildung-17440499.html> [letzter Zugriff am 25.09.2021]; Adler, Sabine: Putins Ukraine-Artikel löst Angst und Schrecken aus, 26.07.2021, URL: <https://srv.deutschlandradio.de/dlf-audiothek-audio-teilen.3265.de.html?mdm:audio_id=944461> [letzter Zugriff am 25.09.2021].

74 Flierl, Thomas/Müller, Elfriede (Hrsg.): Osteuropa – Schlachtfeld der Erinnerungen, Dietz, Berlin 2010.

75 Ackermann, Felix: Aktualisierte Gewalterfahrung. Litauens Geschichtspolitik und Russlands Ukrainekrieg, in: Osteuropa 66 (2016), H. 3, S. 111–128; Sužiedélis, Saulius: Schwierige Erinnerung. Litauen: Holocaust und Opferkonkurrenz, in: Osteuropa 68 (2018), H. 6, S. 101–113; Bobryk, Adam: Die Fesseln der Geschichte. Erinnerungskonflikte in Litauen, in: Osteuropa 70 (2020), H. 1–2, S. 51–60.

76 Werner, Ana Lena: Antiamerikanismus in Aktion. Linke, Rechte und „Querfront“ zur Ukraine, in: Osteuropa 66 (2016), H. 3, S. 129–138; Wehner, Markus: Putins Kalter Krieg. Wie Russland den Westen vor sich hertreibt, Knaur, München 2016, S. 112–130.

77 Wieder Krieg in Europa? Nicht in unserem Namen!, in: ZEIT Online, 05.12.2014, https://www.zeit.de/politik/2014-12/aufruf-russland-dialog [letzter Zugriff am 21.10.2020]. Zuletzt noch einmal mit ähnlicher Argumentation Bahr, Adelheid u. a. (Hrsg.): Warum wir Frieden und Freundschaft mit Russland brauchen, Westend, Frankfurt am Main 2018; Platzeck, Matthias: Wir brauchen eine neue Ostpolitik. Russland als Partner, Propyläen, Berlin 2020.

78 Himmelreich, Jörg: Deutsch-Russische Wahlverwandtschaften. Die „Neue Rechte“ (16.10.2017), URL: <https://www.bpb.de/politik/extremismus/rechtsextremismus/256080/deutsch-russische-wahlverwandtschaften-die-neue-rechte> [letzter Zugriff am 23.10.2020]; Lewis, David G.: Russia’s New Authoritarianism. Putin and the Politics of Order, Edinburgh UP, Edinburgh 2020.

79 Pörzgen, Emma: Moskau fest im Blick. Die deutschen Medien und die Ukraine, in: Osteuropa 64 (2014), H. 5–6, S. 293–310; Osipian, Aleksandr: World War II Memory Politics in Russia and Ukraine and their Uses during the Conflict in the Donbass, in: Amacher: Official History (wie Anm. 39), S. 267–290.

80 Friedenssicherung statt Expansionsbelohnung, URL: <https://www.zeit.de/politik/2014-12/aufruf-friedenssicherung-statt-expansionsbelohnung> [letzter Zugriff am 14.10.2020].

81 Snyder, Timothy: Bloodlands. Europa zwischen Hitler und Stalin, Beck, München 2011.

82 Berkhoff, Karel C.: Harvest of Despair. Life and Death in Ukraine under Nazi Rule, Harvard UP, Cambridge, MA 2004; Penter, Tanja: Kohle für Stalin und Hitler. Arbeiten und Leben im Donbass 1929 bis 1953, Klartext, Essen 2011; Schlögel, Karl: Entscheidung in Kiew. Ukrainische Lektionen, Hanser, München 2015; Kellmann, Klaus: Dimensionen der Mittäterschaft. Die europäische Kollaboration mit dem Dritten Reich, Böhlau, Wien 2019.

83 Breitinger, Matthias: Auch Unterhaltung ist politisch, in: ZEIT Online, 15.05.2016, URL: <https://www.zeit.de/kultur/musik/2016-05/eurovision-song-contest-kommentar> [letzter Zugriff am 21.10.2020].

84 Peters, Florian: Roter Mohn statt Rotem Stern. „Entkommunisierung“ der Geschichtskultur in der Ukraine, in: Osteuropa 66 (2016), H. 3, S. 59–77; Simon, Gerhard: Good Bye, Lenin. Die Ukraine verbietet kommunistische Symbole, in: Osteuropa 66 (2016), H. 3, S. 79–94; Törnquist-Plewa, Barbara/Yurchuk, Yuliya: Memory Politics in Contemporary Ukraine. Reflections from the Postcolonial Perspective, in: Memory Studies 12 (2019), H. 6, S. 699–720.

85 Der ganz im Brüsseler Bürokratensprech verfasste Entschließungsantrag ist mit seiner Fülle historischer Klischees und basaler Generalaussagen abrufbar, URL: <https://www.europarl.europa.eu/doceo/document/TA-9-2019-0021_DE.html> [letzter Zugriff am 23.10.2020].

86 Vgl. dazu schon König, Helmut u. a. (Hrsg.): Europas Gedächtnis. Das neue Europa zwischen nationalen Erinnerungen und gemeinsamer Identität, transcript, Bielefeld 2008.

87 Seit 2011 wird der 23. August – der Tag, an dem der Teufelspakt der beiden Diktatoren 1939 unterzeichnet worden war – als europäischer Gedenktag für die zahllosen Opfer der durch „Staatsterror und Massenmord geprägten totalitären Systeme“ begangen. Jenseits Ostmitteleuropas findet er bislang nur wenig Resonanz. Das soll durch den neuen Entschließungsantrag verändert werden. Vgl. Troebst, Stefan: Der 23. August als euroatlantischer Gedenktag? in: Kaminsky, Anna u. a. (Hrsg:): Der Hitler-Stalin-Pakt 1939 in den Erinnerungskulturen der Europäer, Wallstein, Göttingen 2011, S. 431–467.

88 Vgl. dazu Leggewie, Claus: Schlachtfeld Europa. Transnationale Erinnerung und europäische Identität, in: Bieber, Christoph/Drechsel, Benjamin/Lang, Anne-Katrin (Hrsg.): Kultur im Konflikt. Claus Leggewie Revisited, transcript, Bielefeld 2010, S. 29–44; Feindt, Gregor u. a. (Hrsg.): Europäische Erinnerung als verflochtene Erinnerung. Vielstimmige und vielschichtige Vergangenheitsdeutungen jenseits der Nation, Wallstein, Göttingen 2014; Assmann, Aleida: Der europäische Traum. Vier Lehren aus der Geschichte, Beck, München 2020; Dorn, Lena/Nekula, Marek/Smyčka, Václav (Hrsg.): Zwischen nationalen und transnationalen Erinnerungsnarrativen in Zentraleuropa, De Gruyter, Berlin u. a. 2021.

89 Vgl. bes. Geyer, Michael/Fitzpatrick, Sheila (Hrsg.): Beyond Totalitarianism. Stalinism and Nazism compared, Cambridge UP, Cambridge, MA 2009.

90 Das L.I.S.A.-Wissensschaftsportal der Gerda-Henkelstiftung (URL: <https://lisa.gerda-henkel-stiftung.de/>) hat zum EU-Erschließungsantrag seit November 2019 ausführliche Interviews mit Anke Hilbrenner, Alexej Miller, Jost Dülffer und Gregor Feindt geführt. Darin werden die Argumente für und gegen eine EU-weite, unifizierende Gedenkpolitik anschaulich thematisiert [letzter Zugriff am 26.10.2020].

91 Naryschkin, Sergey: Der UdSSR-Beschluss über den Nichtangriffspakt mit Deutschland gründete sich auf genaue nachrichtendienstliche Erkenntnisse, URL: <https://russische-botschaft.ru/de/2019/09/03/sergey-naryschkin-der-vorsitzende-der-russischen-historischen-gesellschaft-der-udssr-beschluss-ueber-den-nichtangriffspakt-mit-deutschland-gruendete-sich-auf-genaue-nachrichtendienstliche-erkenntnis/> [letzter Zugriff am 26.10.2020].

92 Kolesnikov: Erinnerung (wie Anm. 21), S. 11 u. S. 17, Kurilla, Ivan: Nationalizing Russian (War) Memory since 2014, in: PONARS Eurasian Policy Memo No. 459, July 2020, S. 1–7, hier S. 5.

93 Polen bestellt russischen Botschafter ein, in: ZEIT Online, 28.12.2019, URL: <https://www.zeit.de/politik/ausland/2019-12/wladimir-putin-polen-russland-botschafter-antisemitismus-hitler> [letzter Zugriff am 23.10.2020].

94 Bachmann, Klaus: Viel Feind, viel Ehr. Geschichtspolitik und Außenpolitik in Polen, in: Osteuropa 68 (2018), H. 3–5, S. 413–434; Saryusz-Wolska, Magdalena/Stach, Sabine/Stoll, Katrin: Verordnete Geschichte. Nationalistische Narrative in Polen, in: Osteuropa 68 (2018), H. 3–5, S. 447–464.

95 Stahnke, Jochen: Auschwitz-Gedenken in Israel. Eine Bühne für Putin, in: Frankfurter Allgemeine Zeitung, 22.01.2020, URL: <https://www.faz.net/aktuell/politik/auschwitz-erinnerung-in-israel-eine-buehne-fuer-putin-16595114.html> [letzter Zugriff am 23.10.2020]; Jad Vaschem bedauert Fehler, in: ZEIT Online, 03.02.2020, URL: <https://www.zeit.de/gesellschaft/zeitgeschehen/2020-02/holocaust-gedenken-yad-vashem-filme-fehler-polen-russland> [letzter Zugriff am 23.10.2020].

96 Lipman, Marija: Coronavirus statt Kaiserkrönung. Putins Verfassung und die Pandemie, in: Osteuropa 70 (2020), H. 3–4, S. 89–97.

97 Vgl. URL: <https://nationalinterest.org/feature/vladimir-putin-real-lessons-75th-anniversary-world-war-ii-162982> und URL: <http://en.kremlin.ru/events/president/news/63527> [letzter Zugriff am 23.10.2020].

98 Vgl. URL: <https://russische-botschaft.ru/ru/2020/06/19/statya-vladimira-putina-75-let-velikoy/> und URL: <https://russische-botschaft.ru/de/2020/06/19/75-jahrestag-des-grossen-sieges-gemeinsame-verantwortung-vor-geschichte-und-zukunft/> [letzter Zugriff am 23.10.2020].

99 Zur geschlossenen Kritik der deutschen Historiker_innen an Putins Geschichtsoffensive vgl. bes. Aust, Martin/Hilbrenner, Anke/Obertreis, Julia: Geschichtspolitik braucht Entspannungspolitik, in L.I.S.A. vom 2. Juli 2020, URL: <https://lisa.gerda-henkel-stiftung.de/geschichtspolitik_entspannungspolitik> [letzter Zugriff am 24.10.2020].

100 So der Begriff von Daniel Koerfer: Deutsch-sowjetische Beutepartnerschaft. Zweiter Weltkrieg – Kriegsbeginn 1939, in: Frankfurter Allgemeine Zeitung, 17.09.2009, S. 33.

101 Möller, Horst u. a. (Hrsg.): Die Tragödie Europas. Von der Krise des Jahres 1939 bis zum Angriff auf die UdSSR, De Gruyter Oldenbourg, München 2013; Moorhouse, Roger: The Devils’ Alliance. Hitler’s Pact with Stalin, 1939–1941, Basic Books, New York, NY 2014; Weber, Claudia: Der Pakt. Stalin, Hitler und die Geschichte einer mörderischen Allianz 1939–1941, Beck, München 2019.

102 Diese Sicht geht darüber hinweg, dass sich auch Moskau bemühte, die schon zuvor während der 1920er Jahre praktizierte Zusammenarbeit mit Deutschland weiter fortzusetzen und deshalb dem nationalsozialistischen Berlin immer wieder Offerten unterbreitete.

103 Zarusky, Jürgen/Zückert, Martin (Hrsg.): Das Münchener Abkommen von 1938 in europäischer Perspektive, Oldenbourg, München 2013; Bouverie, Tim: Appeasing Hitler. Chamberlain, Churchill and the Road to War, Bodley Head, New York, NY 2019; Caquet, P. E.: The Bell of Treason. The 1938 Munich Agreement in Czechoslovakia, Other Press, New York, NY 2019. Ironischerweise mussten sich die selbsternannten „Russland-Versteher“ nach der Annexion der Krim den Vorwurf gefallen lassen, eine von Naivität und Ignoranz geprägte *Appeasement*-Politik zu betreiben. Vgl. z. B. Kohler, Berthold: Ukraine-Konflikt. Appeasement 2.0., in: Frankfurter Allgemeine Zeitung, 20.11.2014, URL: <https://www.faz.net/aktuell/politik/ausland/europa/ukraine-konflikt-appeasement-2-0-13275444.html> [letzter Zugriff am 27.12.2020].

104 Lehnstaedt, Stephan: Der vergessene Sieg. Der Polnisch-Sowjetische Krieg 1919/20 und die Entstehung des modernen Osteuropas, Beck, München 2019.

105 Perović, Jeronim: Die „Wende“ als „Verrat“. Russland, die Nato-Osterweiterung und das Scheitern der europäischen Sicherheitsordnung, in: ders./Collmer, Peter/Emeliantseva Koller, Ekaterina (Hrsg.): Zerfall und Neuordnung. Die „Wende“ in Osteuropa von 1989/91, Böhlau, Wien 2019, S. 75–102.

106 Felder, Björn M.: Lettland im Zweiten Weltkrieg. Zwischen sowjetischen und deutschen Besatzern 1940–1946, Schöningh, Paderborn 2009; Angermann, Norbert/Brüggemann, Karsten: Geschichte der baltischen Länder, Reclam, Ditzingen 2018, S. 276–292.

107 van Dyke, Carl: The Soviet Invasion of Finland 1939–40, Routledge, London 1997; Spencer, Malcolm L. G.: Stalinism and the Soviet-Finnish War, 1939–40. Crisis Management, Censorship and Control, Palgrave Macmillan,London 2018.

108 Moorhouse, Roger: First to Fight. The Polish War 1939, Bodley Head, London 2019.

109 Vgl. dazu Nesselrodt, Markus: Dem Holocaust entkommen. Polnische Juden in der Sowjetunion, 1939–1946, De Gruyter Oldenbourg, München 2019.

110 Böhler, Jochen/Lehnstaedt, Stephan (Hrsg.): Gewalt und Alltag im besetzten Polen, 1939–1945, Fibre, Osnabrück 2012; Brewing, Daniel: Im Schatten von Auschwitz. Deutsche Massaker an polnischen Zivilisten 1939–1945, WBG, Darmstadt 2016; Gulińska-Jurgiel, Paulina u. a. (Hrsg.): Ends of War. Interdisciplinary Perspectives on Past and New Polish Regions after 1944, Wallstein, Göttingen 2019.

111 Stöver, Bernd: Der Kalte Krieg, 1947–1991. Geschichte eines radikalen Zeitalters, Beck, München 2007.

112 Putins machtpolitisches Wunschdenken widerspricht diametral der Haltung Gorbatschows und seiner Berater, die den Fall der Berliner Mauer als „Ende von Jalta, das Finale für das Stalin’sche Erbe“ begrüßt hatten. Ihnen galt Jalta als Synonym für die gespaltene europäische Nachkriegsordnung, die sie als historischen Irrtum verstanden und deshalb mit ihrer konstruktiven Friedenspolitik überwinden wollten. Vgl. Karner, Stefan u. a.: Die Sowjetunion und Osteuropa 1989. Zur Einleitung, in: dies. (Hrsg.): Der Kreml und die „Wende“ 1989. Interne Analysen der sowjetischen Führung zum Fall der kommunistischen Regime. Dokumente, StudienVerlag, Innsbruck 2014, S. 13–66, hier S. 45 f.

113 Plokhy, Serhii: Yalta. The Price of Peace, Viking Adult, New York, NY 2010.

114 Vladimir Putin: Überfall auf die Sowjetunion. Offen sein, trotz der Vergangenheit, in: ZEIT-Online, 22.06.2021, URL: <https://www.zeit.de/politik/ausland/2021-06/ueberfall-auf-die-sowjetunion-1941-europa-russland-geschichte-wladimir-putin> [letzter Zugriff am 12.11.2021].

115 Schulze Wessel, Martin: Putins Mythen, 26. Juni 2021, URL: <https://www.zeit.de/politik/ausland/2021-06/wladimir-putin-angriff-sowjetunion-martin-schulze-wessel?utm_source=pocket-newtab-global-de-DE> [letzter Zugriff am 24.09.2020]; Fücks, Ralf: Putin vergiftetes Angebot, in: ZEIT-Online, 24.06.2021, URL: <https://www.zeit.de/politik/ausland/2021-06/russland-wladimir-putin-ralf-fuecks-nato-angebot> [letzter Zugriff am 24.09.2021].

116 Hurrelbrink, Peter: Der 8. Mai 1945. Befreiung durch Erinnerung. Ein Gedenktag und seine Bedeutung für das politisch-kulturelle Selbstverständnis in Deutschland, Dietz, Bonn 2005; Siebeck, Cornelia: „Einzug ins verheißene Land“. Richard von Weizsäckers Rede zum 40. Jahrestag des Kriegsendes am 8. Mai 1985, in: Zeithistorische Forschungen/Studies in Contemporary History, Online-Ausgabe, 12 (2015), H 1, S. 161–169, URL: <www.zeithistorische-forschungen.de/1-2015/id%3D5177> [letzter Zugriff am 05.10.2020]; Hammerstein, Katrin/Hofmann, Birgit: „Wir […] müssen die Vergangenheit annehmen“. Richard von Weizsäckers Rede zum Kriegsende 1985, in: Deutschland Archiv (18.12.2015), URL: <www.bpb.de/217619> [letzter Zugriff am 05.10.2020].

123 Für anregende Gespräche zum Thema, wichtige Korrektur- sowie Literaturhinweise und für den Titelbegriff „Cliotherapeut“ danke ich Daniel Weinmann.

117 Stewart: Geschichte (wie Anm. 35), S. 26 f.; Bastrykin sozdal special’nyj otdel dlja bor’by s fal’sifikaciej istorii, in: RBK, 10.09.2020, URL: <https://www.rbc.ru/society/10/09/2020/5f5a49f89a79479246b62ea4> [letzter Zugriff am 31.10.2020].

118 Smyth, Regina: Informationskriege, Oppositionskoordination und die Dumawahl im Jahr 2021, in: Russland-Analysen Nr. 403 (08.06.2021), S. 4–6; Martynow, Kirill: Great Firewall of Russia, in: Dekoder. Russland entschlüsseln, 18.09.2021, URL: <https://www.dekoder.org/de/article/internet-zensur-duma-google-apple-telegram> [letzter Zugriff am 24.09.2021]. Zu den neuen Zensurmaßnahmen findet sich auf der Internetplattform „Dekoder“ auch ein anschauliches Dossier: Alles Propaganda? Russlands Medienlandschaft, URL: <https://www.dekoder.org/de/dossier/medienlandschaft-propaganda-pressefreiheit> [letzter Zugriff am 24.09.2021].

119 Matthews, Owen: Vladimir Putin’s history fetish, in: The Spectator, 20.06.2020, URL: <https://www.spectator.co.uk/article/vladimir-putin-s-history-fetish> [letzter Zugriff am 31.10.2020].

120 Lipman: Coronavirus (wie Anm. 96); Perović, Jeronim: Virus in the System. Russia and the Corona Crisis, in: CSS Analyses in Security Policy, Nr. 266 (July 2020), URL: <https://css.ethz.ch/content/dam/ethz/special-interest/gess/cis/center-for-securities-studies/pdfs/CSSAnalyse266-EN.pdf> [letzter Zugriff am 14.12.2020]; Apuchtina, Julia/Torotscheschnikowa, Tatjana: Covid-19: Augen zu und durch, in: Dekoder. Russland Entschlüsseln, 31.03.2021, URL: <https://www.dekoder.org/de/article/covid-19-uebersterblichkeit-todesfaelle-statistik> [letzter Zugriff am 24.09.2020].

121 Oushakine, Serguei A.: The Patriotism of Despair. Nation, War, and Loss in Russia, Cornell UP, Ithaca, NY 2009.

122 Ėpple: Neudobnoe prošloe, (wie Anm. 15), S. 19. Den Konnex zwischen „losing the present“ und „nationalizing the past“ sowie das damit einhergehende „inflating ‚memory‘ over ‚history‘“ kommt, thematisiert bes. Kurilla: Memory (wie Anm. 92), S. 5 f.

